# Complication rates following ventricular tachycardia ablation in ischaemic and non-ischaemic cardiomyopathies: a systematic review

**DOI:** 10.1007/s10840-021-00948-6

**Published:** 2021-01-29

**Authors:** Wern Yew Ding, Charles M. Pearman, Laura Bonnett, Ahmed Adlan, Shui Hao Chin, Nathan Denham, Simon Modi, Derick Todd, Mark C. S. Hall, Saagar Mahida

**Affiliations:** 1 Department of Cardiac Electrophysiology, Liverpool Heart and Chest Hospital, Liverpool, UK; 2 Liverpool Centre for Cardiovascular Science, Liverpool, UK; 3 Unit of Cardiac Physiology, Institute of Cardiovascular Sciences, Manchester Academic Health Sciences Centre, The University of Manchester, Manchester, UK; 4 Department of Biostatistics, University of Liverpool, Liverpool, UK

**Keywords:** Catheter ablation, Ventricular tachycardia, Structural heart disease, Ischaemic cardiomyopathy, Non-ischaemic cardiomyopathy, Complications, Death, Mortality

## Abstract

**Background:**

Catheter ablation of ventricular tachycardia (VT) is associated with potential major complications, including mortality. The risk of acute complications in patients with ischaemic cardiomyopathy (ICM) and non-ischaemic cardiomyopathy (NICM) has not been systematically evaluated.

**Methods:**

PubMed was searched for studies of catheter ablation of VT published between September 2009 and September 2019. Pre-specified primary outcomes were (1) rate of major acute complications, including death, and (2) mortality rate.

**Results:**

A total of 7395 references were evaluated for relevance. From this, 50 studies with a total of 3833 patients undergoing 4319 VT ablation procedures fulfilled the inclusion criteria (mean age 59 years; male 82%; 2363 [62%] ICM; 1470 [38%] NICM). The overall major complication rate in ICM cohorts was 9.4% (95% CI, 8.1–10.7) and NICM cohorts was 7.1% (95% CI, 6.0–8.3). Reported complication rates were highly variable between studies (ICM I^2^ = 90%; NICM I^2^ = 89%). Vascular complications (ICM 2.5% [95% CI, 1.9–3.1]; NICM 1.2% [95% CI, 0.7–1.7]) and cerebrovascular events (ICM 0.5% [95% CI, 0.2–0.7]; NICM, 0.1% [95% CI, 0–0.2]) were significantly higher in ICM cohorts. Acute mortality rates in the ICM and NICM cohorts were low (ICM 0.9% [95% CI, 0.5–1.3]; NICM 0.6% [95% CI, 0.3–1.0]) with the majority of overall deaths (ICM 75%; NICM 80%) due to either recurrent VT or cardiogenic shock.

**Conclusion:**

Overall acute complication rates of VT ablation are comparable between ICM and NICM patients. However, the pattern and predictors of complications vary depending on the underlying cardiomyopathy.

**Supplementary Information:**

The online version contains supplementary material available at 10.1007/s10840-021-00948-6.

## Introduction

Ventricular tachycardia (VT) is a major cause of sudden death in patients with structural heart disease. While implantable cardiac defibrillators (ICD) reduce the risk of sudden death [1, 2], recurrent ICD shocks due to VT remains an important cause of morbidity and mortality [3]. Catheter ablation is an effective technique for reducing the risk of recurrent VT and ICD therapies in patients with ischaemic and non-ischaemic cardiomyopathies (ICM; NICM) [4–6]. However, VT ablation is associated with a risk of major complications including mortality, and a decision to undertake VT ablation involves balancing these risks against potential benefits in terms of arrhythmia-free survival.

To date, complication rates associated with VT ablation have largely been defined based on data from single-centre studies or multicentre studies with limited sample sizes [7–9]. A comprehensive synthesis of available literature with a focus on procedure-related complications has not been undertaken. Furthermore, despite differing substrates, co-morbidities and ablation strategies, the relative complication rates of VT ablation in patients with ICM and NICM are not fully defined. An in-depth understanding of complication rates has potential implications in terms of patient selection, ablation strategies and informed consent.

The aim of this systematic review was to assess the risk of major acute complications associated with catheter ablation of VT.

## Methods

**Fig. 1:**
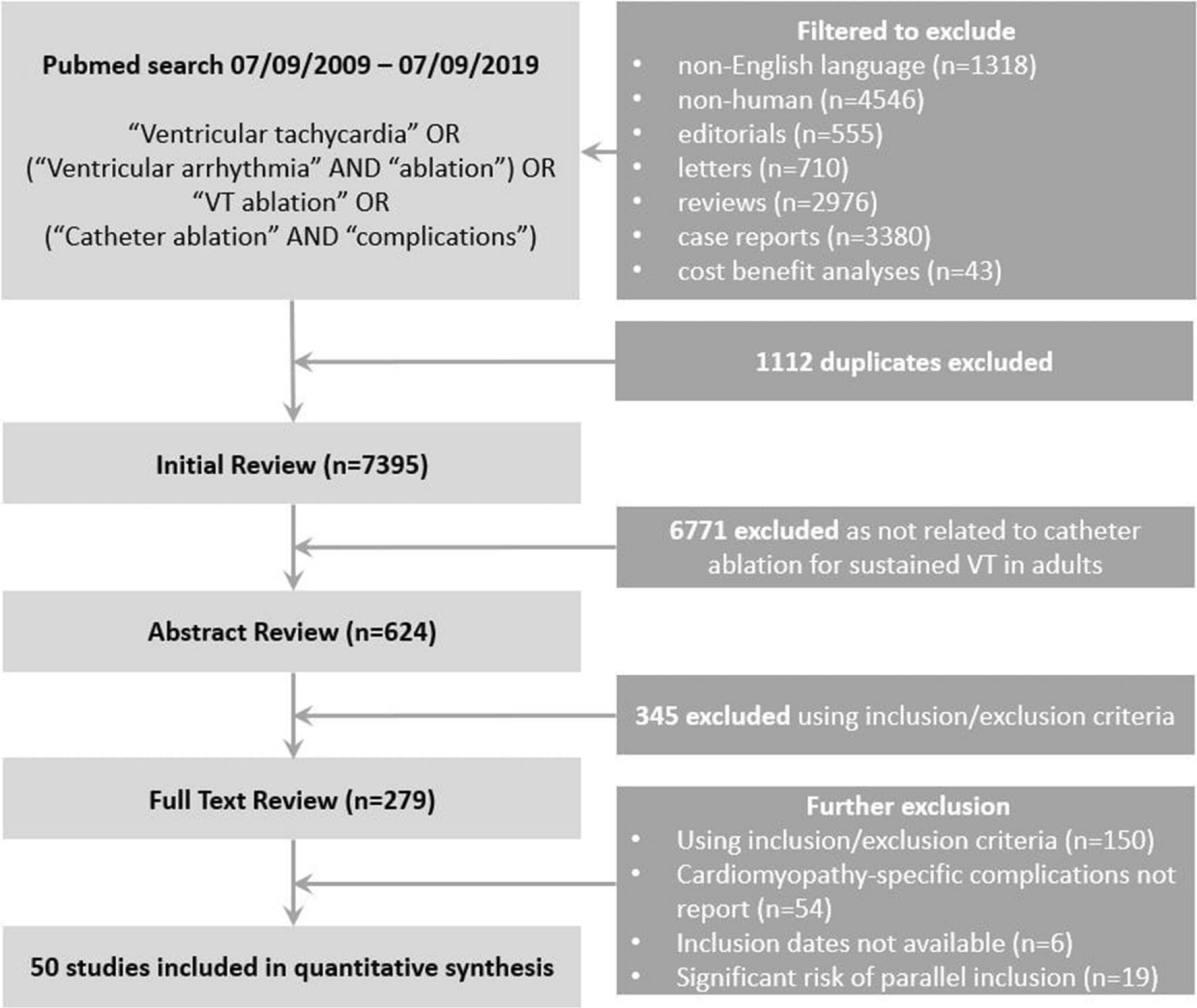
Flow diagram of search strategy

A literature search was performed using PubMed to identify all relevant studies published on catheter ablation of sustained VT in adults with structural heart disease between September 2009 and September 2019. This date range was selected in order to ensure that the findings are more applicable to current clinical practice. The study adhered to the Preferred Reporting Items for Systematic Reviews and Meta-analyses [10] and Meta-analysis of Observational Studies in Epidemiology guidelines for reporting [11]. Inclusion of other relevant studies not identified during our initial search was permitted. A description of our search strategy is summarised in Fig. 1. Detailed search methodology is included in the Supplemental Material.

### Outcome measures

The pre-specified primary outcome measures were the rates of (1) major acute complications, including death, and (2) mortality. To improve specificity and avoid including deaths unrelated to the procedure in these multimorbid cohorts, only deaths within the same admission or 7 days of VT ablation were included. The secondary outcome measure was the rate of specific complications. Acute complications included vascular access-related complications, pericardial effusion, cardiac perforation, need for cardiac surgery, complete atrioventricular block, cerebrovascular accident (CVA) or transient ischaemic attack (TIA), cardiogenic shock or severe pulmonary oedema, lead displacement, venous or arterial thromboembolism, myocardial infarction, other major bleeding, infection requiring antibiotics, phrenic nerve palsy and pneumothorax or haemothorax. Other major bleeding was based on study-specific definitions that were not related to vascular access-related complications, pericardial effusion or cardiac perforation.

### Study selection

Inclusion criteria for studies were as follows: (1) participants aged ≥18 years, (2) ≥95% study participants with VT ablation in the context of structural heart disease, (3) reporting of complications following VT ablation stratified by cardiomyopathy subtype and (4) ≥20 patients undergoing VT ablation. Exclusion criteria for studies were as follows: (1) surgical ablation techniques were used; (2) ≥5% of participants who underwent catheter ablation had congenital heart disease; (3) ≥5% participants underwent catheter ablation of premature ventricular complexes alone; (4) ≥5% participants underwent catheter ablation for ventricular fibrillation; (5) animal models or *in vitro* studies; (6) not published in English; (7) abstracts, review articles, conference proceedings, editorials and meta-analyses; and (8) potential parallel inclusion of the same participants/cohorts from a single centre in multiple studies.

We applied a systematic approach to minimise parallel repetition. In situations where centres had reported patient cohorts in multiple studies, potential parallel inclusion of participants was identified based on the recruitment period. As such, studies with potential parallel inclusion in which the recruitment period was not specified were excluded. For the remainder, those with <20% overlap of the recruitment period were included. Where multiple studies had ≥20% overlap of the recruitment period, only one study was included, prioritising randomised controlled trials (RCTs) over case-control and cohort studies. In the presence of multiple cohort studies only, the study with the largest cohort was prioritised.

### Data extraction and quality assessment

All included manuscripts were assessed independently by two reviewers. Any differences were resolved by consensus. Quality assessment was performed using a list of 8-quality items that addressed both the internal and external validity of a study (Supplemental Material) [12].

### Statistical analysis

Data analysis was performed using R (version 3.6) [13]. Pooled estimates were obtained across studies using a random effects model according to the DerSimonian and Laird method [14]. The I^2^ statistic was used to quantify heterogeneity between studies [15]. Univariable meta-regression was used to evaluate the impact of clinical covariates on major acute complications. A two-sided *p* value of <0.05 was considered statistically significant. Risk of publication bias was assessed using Funnel plot and Egger’s test. Study weighting was assigned using number of participants in each study rather than standard error to avoid excessive weighting of those reporting no complications. Studies with reported events rates of zero were assigned values of 0.005 to avoid dividing by a zero count which would yield a computational error.

## Results

### Study characteristics

**Fig. 2:**
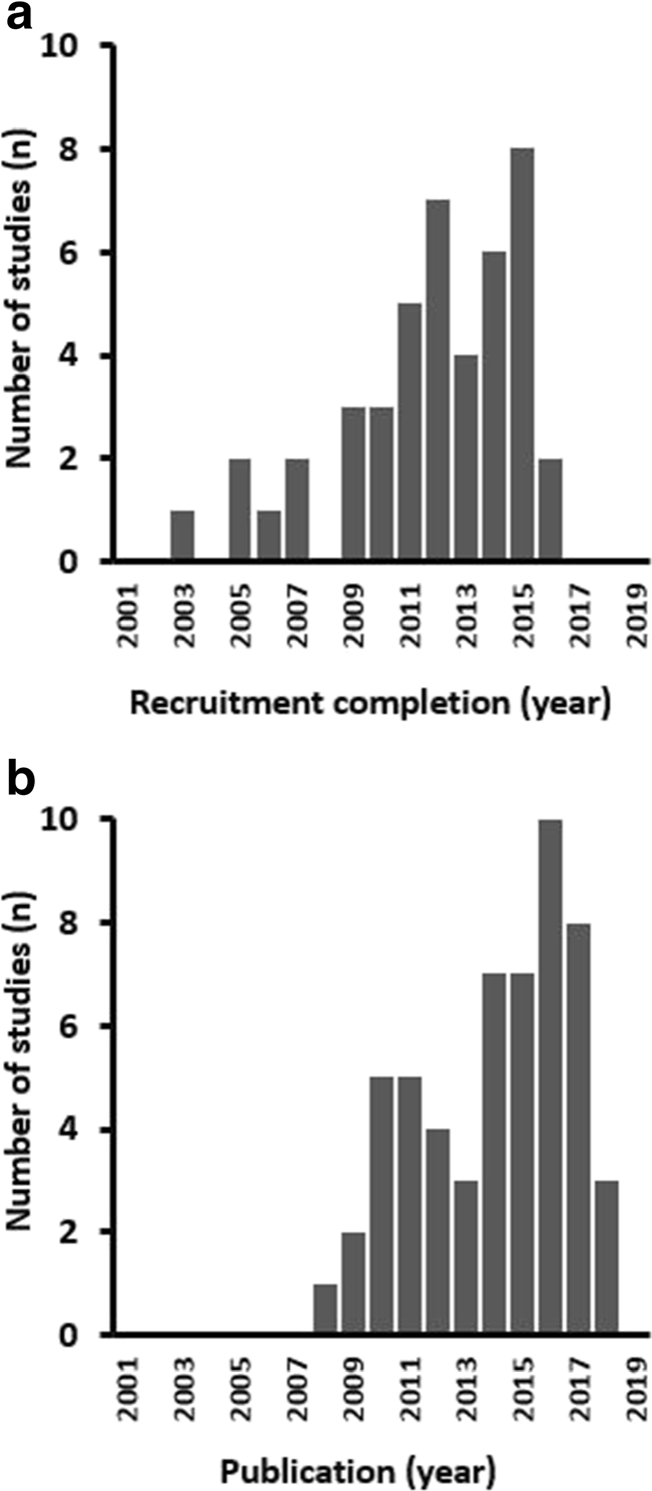
Histograms for distribution of recruitment and publication dates

The initial literature search yielded 7395 citations. After sequential filtering (7116 excluded following title/abstract review; 229 excluded after full text review), 50 studies were included in the final analysis (45 cohort studies, 4 randomised controlled trials and 1 case-control study). Forty-one (82%) were single-centre studies. Overall, the 50 studies included 3833 patients who underwent a total of 4319 VT ablation procedures. Twenty-nine studies included an ICM cohort, while 23 studies included an NICM cohort. Histograms of the final recruitment period and publication dates are included in Fig. 2. Study characteristics are included in Supplemental Table 1.

### Demographics and procedural characteristics

Overall, 82% of study subjects were male (mean age 59 years; mean left ventricular ejection function 37%). Baseline demographics and procedural characteristics of the overall cohort are included in Supplemental Table 2. Data on ICD was available for 43 (86%) studies. In total, 2973/3343 (84.4%) patients from these studies had an ICD either prior to or during the time of ablation. A total of 2363 (62%) patients had ICM, while 1470 (38%) had NICM. Compared to the NICM population, the mean age of the ICM population was higher (65 years vs 51 years) with a lower proportion of female patients (10% vs 29%) and re-do procedures (5% vs 23%). The procedure time was shorter for the ICM population (225 vs 273 min) despite a longer ablation time (39 vs 32 min). Eight percent of ablations in the ICM population involved combined endocardial/epicardial access compared to 45% in the NICM population. ICM and NICM cohort-specific baseline and procedural characteristics are included in Table 1.

**Table 1:** Demographics and procedural characteristics of ICM and NICM cohorts

	ICM					NICM				
	Number of studies	Mean	SD	Minimum	Maximum	Number of studies	Mean	SD	Minimum	Maximum
Males (%)	27	90.1	6.2	71.4	100	23	71.5	15.0	30.6	90.9
Age (years)	29	65.2	5.9	37.0	72.0	23	50.5	9.6	34.0	65.0
LV ejection fraction (%)	28	31.3	4.9	21.0	49.0	19	43.6	10.1	29.0	57.0
Diabetes (%)	12	30.7	10.5	14.3	50.0	5	21.6	17.6	4.3	42.9
Renal failure (%)	6	13.8	8.0	0	22.6	3	21.2	12.8	8.9	34.5
VT storm (%)	17	51.6	35.2	0	100	13	50.3	30.3	17.8	100
NYHA class III or IV (%)	11	32.5	19.1	11.1	65.0	10	27.9	19.6	0	54.8
Prior catheter VT ablation (%)	18	4.5	9.7	0	29.6	16	23.0	27.8	0	100
Procedure time (minutes)	23	225.2	81.2	104.0	453.0	16	273.3	110.0	161.0	480.0
Ablation time (minutes)	15	39.2	31.2	9.0	114.0	11	32.2	17.0	11.0	70.0
Ablation site (%)										
LV only	10	97.5	6.3	80.2	100	10	16.0	35.0	0	100
RV only	10	0.1	0.4	0	1.4	10	83.1	34.7	0	100
Both LV and RV	10	2.4	5.9	0	18.4	10	0.9	2.7	0	8.7
Access site (%)										
Endocardial only	23	92.0	13.8	48.9	100	20	49.7	31.2	0	90.2
Epicardial only	23	0.1	0.3	0	1.3	21	3.7	12.9	0	59.1
Combined endocardial/epicardial	23	8.0	13.8	0	51.1	21	45.0	33.7	0	100
Approach type (%)										
Antegrade only	9	44.0	45.1	0	100	4	3.9	4.6	0	8.7
Retrograde only	9	40.3	44.1	0	97.7	3	52.1	50.1	0	100
Both ante- and retro-grade	9	15.6	32.8	0	100	3	14.6	25.2	0	43.7
Pre-procedural anticoagulation (%)	14	100	0	100	100	4	90.0	20.0	60.0	100

*ICM* ischaemic cardiomyopathy, *LV* left ventricular, *NICM* non-ischaemic cardiomyopathy, *NYHA* New York Heart Association, *RV* right ventricular, *SD* standard deviation, *VT* ventricular tachycardia

### Major complications

Pooled major acute complications are included in Supplemental Table 3. The overall rate of major complication (including death) was 8.8% (95% confidence interval (CI), 7.9–9.7). The most common complications were vascular access-related complications (2.0% [95% CI, 1.6–2.4]) and pericardial effusion (1.4% [95% CI, 1.1–1.8]). However, only a minority of these cases required intervention (vascular: 0.2% [95% CI, 0.1–0.4]; pericardial effusion: 0.9% [95% CI, 0.6–1.1]). The major complication rate was comparable in ICM and NICM cohorts (ICM 9.4% [95% CI, 8.1–10.7] vs NICM 7.1% [95% CI, 6.0–8.3]). Overall significantly more vascular access-related complications and CVA/TIA were reported in ICM cohorts compared to NICM, 2.5% (95% CI, 1.9–3.1) vs 1.2% (95% CI, 0.7–1.7) and 0.5% (95% CI, 0.2–0.7) vs 0.1% (95% CI, 0–0.2), respectively. Specific complications in ICM and NICM cohorts are included in Table 2.

**Table 2:** Major acute complications in ICM and NICM cohorts

	ICM (n = 29*)		NICM (*n* = 23*)	
	% Pooled complication rate (95% CI)	I^2^ statistic (%)	% Pooled complication rate (95% CI)	I^2^ statistic (%)
Any major acute complications	9.40 (8.07–10.73)^+^	90	7.14 (5.95–8.33)^§^	89
Death	0.92 (0.51–1.33)^+^	0	0.63 (0.26–1.00)^§^	0
Any vascular access-related complications	2.53 (1.92–3.14)	60	1.16 (0.67–1.65)	8
Vascular access-related complications requiring intervention	0.21 (0.03–0.39)	0	0.23 (0.01–0.44)	0
Any pericardial effusion	1.58 (1.08–2.07)	31	1.27 (0.76–1.78)	15
Pericardial effusion requiring drainage	0.67 (0.35–0.99)	0	1.16 (0.67–1.65)	6
Need for cardiac surgery	0.25 (0.05–0.45)	0	0.39 (0.10–0.68)	0
Complete AV block	0.54 (0.25–0.83)	0	0.17 (0–0.36)	0
CVA or TIA	0.46 (0.19–0.73)	0	0.06 (0–0.17)	0
Cardiac perforation	0.17 (0.01–0.34)	0	0.28 (0.04–0.52)	0
Cardiogenic shock or severe pulmonary oedema	0.42 (0.16–0.67)	0	0.12 (0–0.27)	0
Lead displacement	0.38 (0.14–0.62)	0	0.01 (0–0.04)	0
Venous or arterial thromboembolism	0.05 (0–0.13)	0	0.44 (0.14–0.75)	0
MI	0.09 (0–0.21)	0	0.23 (0.01–0.44)	0
Other major bleeding	0.13 (0–0.27)	0	0.06 (0–0.17)	0
Infection requiring antibiotics	0.09 (0–0.21)	0	0.01 (0–0.04)	0
Phrenic nerve palsy	0.05 (0–0.13)	0	0.12 (0–0.27)	0
Pneumothorax or haemothorax	0.13 (0–0.27)	0	0.01 (0–0.04)	0

*Unless otherwise stated

^+^*n = 24*

^§^*n = 19*

*AV* atrioventricular, *CI* confidence interval, *CVA* cerebrovascular accident, *ICM* ischaemic cardiomyopathy, *MI* myocardial infarction, *NICM* non-ischaemic cardiomyopathy, *TIA* transient ischaemic attack

### Acute mortality

**Fig. 3:**
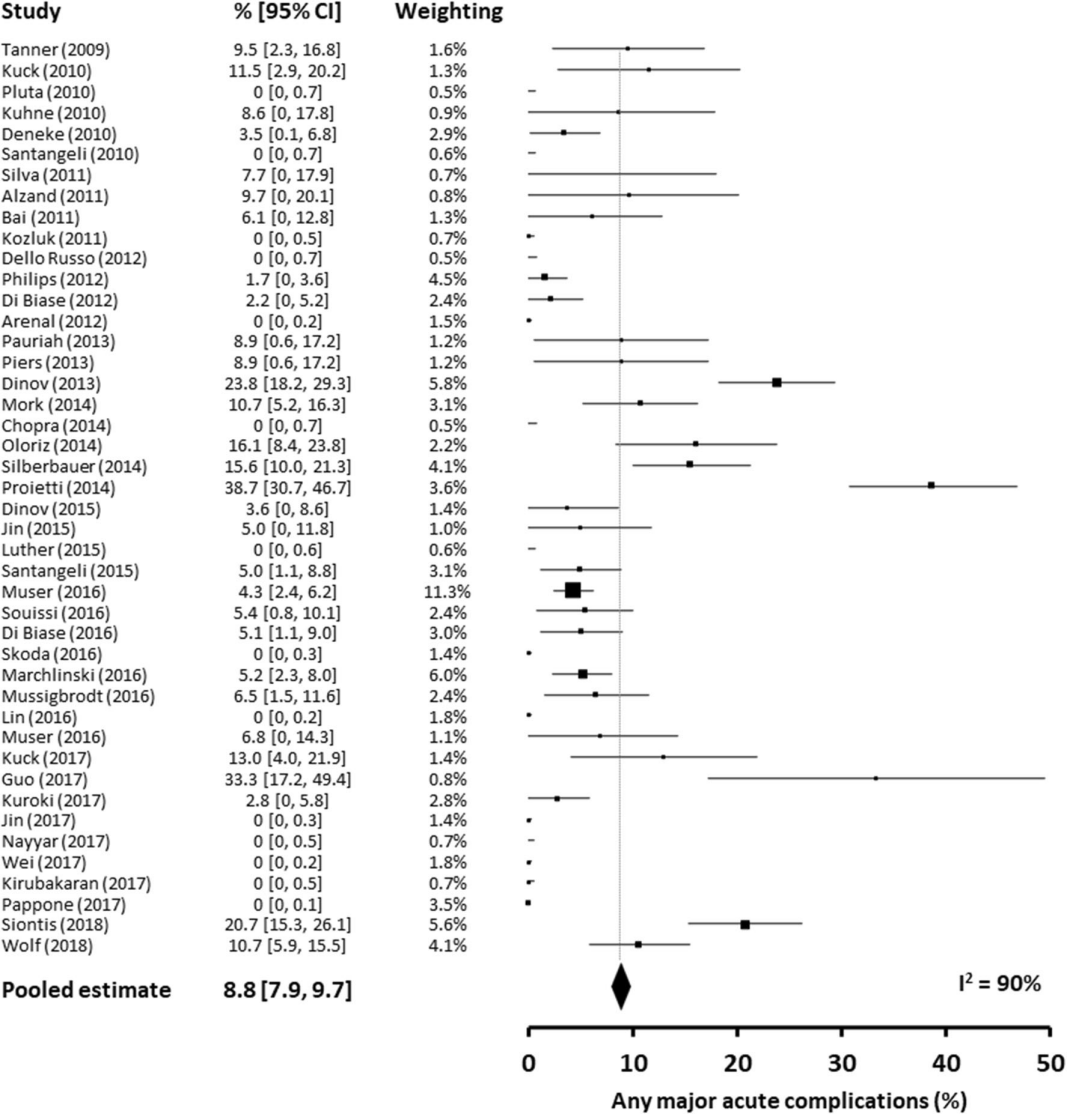
Forest plot of acute major complications from each study

The acute mortality rate (mortality rate during the same admission or within 7 days of VT ablation) was 0.9% (95% CI, 0.6–1.1). Mortality rates in the ICM and NICM cohorts were comparable (ICM 0.9% [95% CI, 0.5–1.3] vs NICM 0.6% [95% CI, 0.3–1.0]). The mode of death was available in 20 of the 21 reported ICM deaths (9 [45%] recurrence of ventricular arrhythmia, 6 [30%] cardiogenic shock, 1 [5%] hepato-renal failure, 1 [5%] septic shock, 1 [5%] pneumonia, 1 [5%] acute respiratory distress syndrome, 1 [5%] left ventricular perforation). The mode of death was available in 10 of 12 of the reported NICM deaths (7 [70%] cardiogenic shock, 1 [10%] recurrence of ventricular arrhythmia, 1 [10%] left ventricular perforation, 1 [10%] pulmonary embolism). A forest plot of the major complications is included in Fig. 3.

### Heterogeneity

We observed significant heterogeneity between studies in terms of reported overall complication rates (I^2^ = 90%, Fig. 3). When considering ICM and NICM separately, the degree of heterogeneity in complication rates remained high for each subtype (ICM I^2^ = 90; NICM I^2^ = 89). Of note, when considering RCTs only, we also observed a moderate to high degree of heterogeneity for overall complications (I^2^ = 45%) and common complications (vascular access-related complications [I^2^ = 24%], pericardial effusion [I^2^ = 59%] and lead displacement [I^2^ = 60%]). Similarly, there was a high degree of heterogeneity for overall complications (I^2^ = 91%) when we limited our analysis to studies with >100 patients. We did not observe significant heterogeneity between studies (I^2^ = 0%) when considering acute mortality alone.

### Temporal trends of complication rates

In order to determine whether there was a temporal trend in complication rates, we compared studies reported in the first 5 years of our inclusion period (2009–2014) to studies reported in the second 5 years (2015–2019). Despite broadly comparable baseline and procedural characteristics, no significant difference in major acute complications was seen between these two time periods in either ICM or NICM cohorts (ICM: 2009–2014, 9.7% [95% CI, 7.5–11.8] vs 2014–2019, 9.2% [95% CI, 7.4–11.0]; NICM: 2009–2014, 6.3% [95% CI, 3.8–8.9] vs 2014–2019, 7.4% [95% CI, 6.1–8.8]) (Supplemental Tables 4–7).

### Study quality and risk of bias

**Fig. 4:**
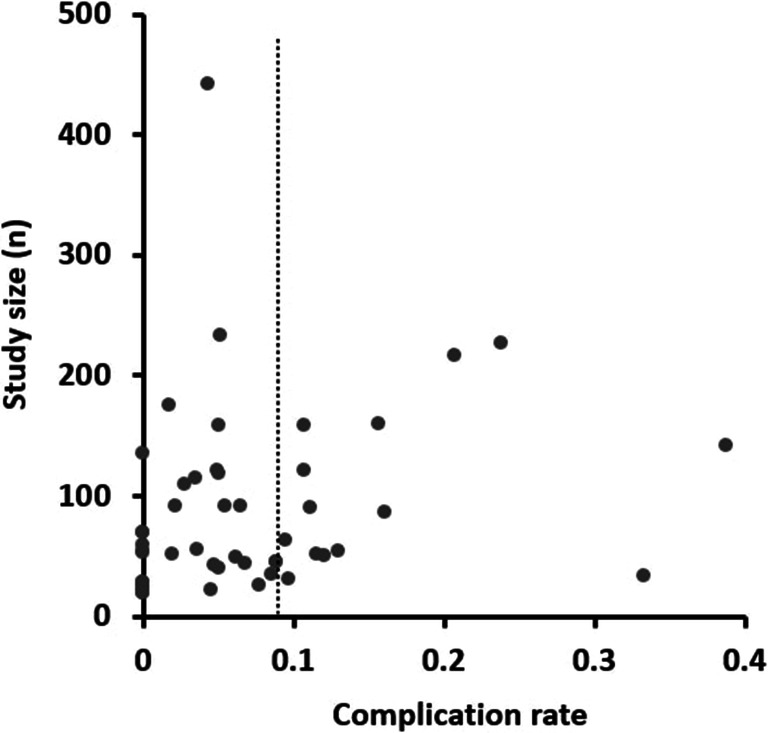
Funnel plot of studies

The quality of studies included was variable using the pre-defined quality assessment criteria (Supplemental Materials): 5 (10%) met all eight criteria; 27 (54%) met at least six criteria; and 11 (22%) met four or less criteria. A detailed description of the study population was provided in 42 studies (84%), and complication rates were prospectively collected in 24 studies (48%). Funnel plot and Egger’s test were used to assess the risk of publication bias (Fig. 4). The Funnel plot was drawn using *n* on the vertical axis as the more commonly used standard error was unduly influenced by small studies report zero complication rates [16]. While Egger’s test was significant (*z* = 13.2103, *p* < 0.0001) suggesting possible publication bias, the plot was symmetrical on visual assessment. Furthermore, it is likely that confounding factors such as case mix and experience may have influenced the relationship between study size and reported complication rate separate to publication bias.

### Sensitivity analyses

In keeping with our primary analysis, when only prospective studies (*n* = 24; 2135 patients) and RCTs were included (*n* = 4; 356 patients), the rates of major complications were 6.5% (95% CI, 4.1–10.2) and 8.5% (95% CI, 3.1–13.9), respectively. The results are included in Supplemental Tables 8 and 9.

## Discussion

The main findings from this systematic analysis which included 4319 VT ablation procedures are as follows: (1) the risk of mortality in the acute phase post-VT ablation is low (<1%) in both ICM and NICM cohorts, with approximately three quarters of the reported cases attributed to recurrent ventricular arrhythmias or cardiogenic shock; (2) the overall major acute complications rates following VT ablation were comparable in ICM and NICM cohorts; (3) the profile of complications differs between ICM and NICM, with vascular access-related complications and CVA/TIA occurring with a significantly higher frequency in ICM cohorts; and (4) there was a high degree of variability in complication rates between studies.

The mortality rate in the acute phase post-procedure was low (<1%), with the predominant causes of death being recurrent ventricular arrhythmias and cardiogenic shock (75–80%). Amongst those patients with recurrent ventricular arrhythmias, mortality is attributable to a failure to alter the trajectory of the disease process rather than a direct procedure-related complication. In the ICM cohort, recurrence of VT accounted for close to half of the mortality cases. Understanding the relationship between VT ablation and cardiogenic shock is more complex. On the one hand, cardiogenic shock may represent the endpoint of the natural trajectory of the disease, while on the other, haemodynamic instability during an ablation procedure may have an adverse effect on cardiac pump function. Distinguishing between procedure-related complications and disease trajectory has important implications in terms of patient consent.

The variability in reported complication rates is a potentially important finding of this study. The complication rates ranged from 0 to 38.7%. Given the variability in study quality, a proportion of the observed heterogeneity is likely to be attributable to differences in complication reporting and the influence of smaller studies with zero complication rates. However, a significant proportion in the variability is likely to be accounted by true variation in complication rates. This point is underscored by the fact that we observed moderate to high degree of heterogeneity even when considering prospective studies and RCTs alone. There are a number of potential explanations for variability. Firstly, we observed significant variation in patient baseline characteristics between studies. Secondly, procedural characteristics including access route and ablation time varied significantly between studies. Thirdly, there is potential variation in the level of expertise between different centres. Fourthly, the timing of VT ablation is likely to vary between centres. Finally, ablation strategies were highly variable between studies. For instance, VT ablation techniques guided by advanced image-integration techniques, more refined electrogram detection and ablation strategies that avoid VT induction are predicted to reduce procedure-related risk [17]. Overall, complication rates are likely to vary significantly due to a complex interaction between patient-specific and operator-specific factors. Our findings highlight the point that reliance on individual-centre or individual-study data to define complication rates is related with important limitations.

While the overall risk of complications was comparable between ICM and NICM cohorts, as discussed above, we observed a high degree of variability in complication rates between studies. Therefore, the comparisons between ICM and NICM in terms of absolute complication rates should be interpreted with caution. Our results do however indicate that the profile of complications between ICM and NICM is different. Vascular access-related complications and CVA/TIA were twofold and eightfold higher amongst ICM patients, respectively. Of note, however, only a small minority of patients required vascular interventions. This observation is in keeping with a higher prevalence of atherosclerotic vascular disease in ICM patients. Our findings underscore the importance of careful selection of the access route in ICM patients undergoing VT ablation.

Overall, the findings of present study indicate that the mortality associated with VT ablation is low. Furthermore, the need for further interventions due to complications remains low. The study also highlights the challenges associated with defining complication rates following VT ablation. Generalisation of results from individual studies is associated with potential challenges, and individualised risk stratification strategies are needed. Our findings highlight the need for potential patient-level meta-analyses in the future to better understand complication rates in different subsets of patients undergoing VT ablation.

## Limitations

One of the inherent limitations of this systematic analysis which is inclusive of a range of studies is a risk of publication, selection and reporting bias. We attempted to minimise these effects by employing a rigorous search strategy, using a strict inclusion and exclusion criteria and excluding studies with very small cohorts (*n* < 20). Furthermore, there was a significant variability in the quality scores of studies. The active decision to maintain inclusion of studies with low quality scores was supported by Stein et al. that found no relationship between these scores and study outcomes [18]. Patient selection, the timing of VT ablation, the specific ablation strategy and evolving technologies have an important impact on potential complication rates. Due to a lack of detailed data, we were unable to define the impact of these variables on complication rates. It is important to note that this study did not involve direct comparisons between ICM and NICM cohorts; therefore, conclusions regarding relative complication rates should be drawn with caution. Finally, the results should be interpreted in the context of the studies cited, and it is worth highlighting that VT ablation is a procedure where operator skill and patient selection may be limited.

## Supplementary information

ESM 1 (DOCX 113 kb)

## Data Availability

The data that support the findings of this study are available from the corresponding author, WYD, upon reasonable request.

## References

[1] Moss AJ, Zareba W, Hall WJ, Klein H, Wilber DJ, Cannom DS, Daubert JP, Higgins SL, Brown MW, Andrews ML (2002). Prophylactic implantation of a defibrillator in patients with myocardial infarction and reduced ejection fraction. N Engl J Med.

[2] Bardy GH, Lee KL, Mark DB, Poole JE, Packer DL, Boineau R, Domanski M, Troutman C, Anderson J, Johnson G, McNulty SE, Clapp-Channing N, Davidson-Ray LD, Fraulo ES, Fishbein DP, Luceri RM, Ip JH (2005). Amiodarone or an implantable cardioverter-defibrillator for congestive heart failure. N Engl J Med.

[3] Proietti R, Labos C, Davis M, Thanassoulis G, Santangeli P, Russo V, di Biase L, Roux JF, Verma A, Natale A, Essebag V (2015). A systematic review and meta-analysis of the association between implantable cardioverter-defibrillator shocks and long-term mortality. Can J Cardiol.

[4] Fernandez-Armenta J, Andreu D, Penela D, Trucco E, Cipolletta L, Arbelo E (2014). Sinus rhythm detection of conducting channels and ventricular tachycardia isthmus in arrhythmogenic right ventricular cardiomyopathy. Heart Rhythm.

[5] Kuck K-H, Schaumann A, Eckardt L, Willems S, Ventura R, Delacretaz E (2010). Catheter ablation of stable ventricular tachycardia before defibrillator implantation in patients with coronary heart disease (VTACH): a multicentre randomised controlled trial. Lancet.

[6] Sapp JL, Wells GA, Parkash R, Stevenson WG, Blier L, Sarrazin J-F, Thibault B, Rivard L, Gula L, Leong-Sit P, Essebag V, Nery PB, Tung SK, Raymond JM, Sterns LD, Veenhuyzen GD, Healey JS, Redfearn D, Roux JF, Tang AS (2016). Ventricular tachycardia ablation versus escalation of antiarrhythmic drugs. N Engl J Med.

[7] Barra S, Begley D, Heck P, Turner I, Agarwal S (2015). Ablation of ventricular tachycardia in the very elderly patient with cardiomyopathy: how old is too old?. Can J Cardiol.

[8] Mahapatra S, Tucker-Schwartz J, Wiggins D, Gillies GT, Mason PK, McDaniel G, LaPar DJ, Stemland C, Sosa E, Ferguson JD, Bunch TJ, Ailawadi G, Scanavacca M (2010). Pressure frequency characteristics of the pericardial space and thorax during subxiphoid access for epicardial ventricular tachycardia ablation. Heart Rhythm.

[9] Bunch TJ, Darby A, May HT, Ragosta M, Lim DS, Taylor AM, DiMarco JP, Ailawadi G, Revenaugh JR, Weiss JP, Mahapatra S (2012). Efficacy and safety of ventricular tachycardia ablation with mechanical circulatory support compared with substrate-based ablation techniques. Europace.

[10] Moher D, Liberati A, Tetzlaff J, Altman DG (2009). Preferred reporting items for systematic reviews and meta-analyses: the PRISMA statement. PLoS Med.

[11] Stroup DF, Berlin JA, Morton SC, Olkin I, Williamson GD, Rennie D, Moher D, Becker BJ, Sipe TA, Thacker SB (2000). Meta-analysis of observational studies in epidemiology: a proposal for reporting. Meta-analysis of Observational Studies in Epidemiology (MOOSE) group. JAMA.

[12] Gupta A, Perera T, Ganesan A, Sullivan T, Lau DH, Roberts-Thomson KC, Brooks AG, Sanders P (2013). Complications of catheter ablation of atrial fibrillation: a systematic review. Circ Arrhythm Electrophysiol.

[13] Core Team R (2019). R: a language and environment for statistical computing.

[14] DerSimonian R, Laird N (1986). Meta-analysis in clinical trials. Control Clin Trials.

[15] Higgins JPT, Thompson SG (2002). Quantifying heterogeneity in a meta-analysis. Stat Med.

[16] Sterne JA, Egger M (2001). Funnel plots for detecting bias in meta-analysis: guidelines on choice of axis. J Clin Epidemiol.

[17] Fernandez-Armenta J, Soto-Iglesias D, Silva E, Penela D, Jáuregui B, Linhart M, Bisbal F, Acosta J, Fernandez M, Borras R, Villuendas R, Cano L, Guasch E, Mont L, Berruezo A (2020). Safety and outcomes of ventricular tachycardia substrate ablation during sinus rhythm: a prospective multicenter registry. JACC Clin Electrophysiol.

[18] Stein K, Dalziel K, Garside R, Castelnuovo E, Round A (2005). Association between methodological characteristics and outcome in health technology assessments which included case series. Int J Technol Assess Health Care.

