# Supplementary material for: Complication rates following ventricular tachycardia ablation in ischaemic and non-ischaemic cardiomyopathies: a systematic review
Source: J Interv Card Electrophysiol. 2021 Jan 29;63(1):59–67. doi: 10.1007/s10840-021-00948-6 (PMC8755671; doi:10.1007/s10840-021-00948-6)
Supplement: Supplementary file 1 — (DOCX 113 kb) [file 10840_2021_948_MOESM1_ESM.docx]

SUPPLEMENTAL MATERIAL

**Complication rates following Ventricular Tachycardia Ablation in Ischaemic and Non-Ischaemic Cardiomyopathies; A Systematic Review**

Short title: Complications of VT ablation in ICM vs NICM

Wern Yew Ding^1,2^; Charles M Pearman, PhD^1,3^; Laura Bonnett^4^; Ahmed Adlan, PhD^1^; Shui Hao Chin, PhD^1^; Nathan Denham^3^; Simon Modi, MD^1^; Derick Todd, MD^1^; Mark CS Hall, MD^1^; Saagar Mahida, MD, PhD^1,2^

1. Department of Cardiac Electrophysiology, Liverpool Heart and Chest Hospital, Liverpool, UK
2. Liverpool Centre for Cardiovascular Science, Liverpool, UK
3. Unit of Cardiac Physiology, Institute of Cardiovascular Sciences, Manchester Academic Health Sciences Centre, The University of Manchester, Manchester, UK
4. Department of Biostatistics, University of Liverpool, Liverpool, UK

Corresponding author:

Dr Wern Yew Ding Department of Cardiac Electrophysiology,

Liverpool Heart and Chest Hospital,

United Kingdom L14 3PE

Telephone 0151 600 1606

Email [dwyew@hotmail.com](mailto:wding@nhs.net)

# Search strategy (Pubmed)

1. ("tachycardia, ventricular"[MeSH Terms] OR ("tachycardia"[All Fields] AND "ventricular"[All Fields]) OR "ventricular tachycardia"[All Fields] OR ("ventricular"[All Fields] AND "tachycardia"[All Fields])) OR (("heart ventricles"[MeSH Terms] OR ("heart"[All Fields] AND "ventricles"[All Fields]) OR "heart ventricles"[All Fields] OR "ventricular"[All Fields]) AND ("arrhythmias, cardiac"[MeSH Terms] OR ("arrhythmias"[All Fields] AND "cardiac"[All Fields]) OR "cardiac arrhythmias"[All Fields] OR "arrhythmia"[All Fields]) AND ablation[All Fields])
   1. Results: 41761 references
      1. From 07/09/2009 to 07/09/2019: 13968 remaining, 27793 removed
         - ("tachycardia, ventricular"[MeSH Terms] OR ("tachycardia"[All Fields] AND "ventricular"[All Fields]) OR "ventricular tachycardia"[All Fields] OR ("ventricular"[All Fields] AND "tachycardia"[All Fields])) OR (("heart ventricles"[MeSH Terms] OR ("heart"[All Fields] AND "ventricles"[All Fields]) OR "heart ventricles"[All Fields] OR "ventricular"[All Fields]) AND ("arrhythmias, cardiac"[MeSH Terms] OR ("arrhythmias"[All Fields] AND "cardiac"[All Fields]) OR "cardiac arrhythmias"[All Fields] OR "arrhythmia"[All Fields]) AND ablation[All Fields]) AND ("2009/09/07"[PDAT] : "2019/09/07"[PDAT])
      2. English only: 13211 remaining, 757 removed
         - ("tachycardia, ventricular"[MeSH Terms] OR ("tachycardia"[All Fields] AND "ventricular"[All Fields]) OR "ventricular tachycardia"[All Fields] OR ("ventricular"[All Fields] AND "tachycardia"[All Fields])) OR (("heart ventricles"[MeSH Terms] OR ("heart"[All Fields] AND "ventricles"[All Fields]) OR "heart ventricles"[All Fields] OR "ventricular"[All Fields]) AND ("arrhythmias, cardiac"[MeSH Terms] OR ("arrhythmias"[All Fields] AND "cardiac"[All Fields]) OR "cardiac arrhythmias"[All Fields] OR "arrhythmia"[All Fields]) AND ablation[All Fields]) AND (("2009/09/07"[PDAT] : "2019/09/07"[PDAT]) AND English[lang])
      3. Human studies only: 9584 remaining, 3627 removed
         - ("tachycardia, ventricular"[MeSH Terms] OR ("tachycardia"[All Fields] AND "ventricular"[All Fields]) OR "ventricular tachycardia"[All Fields] OR ("ventricular"[All Fields] AND "tachycardia"[All Fields])) OR (("heart ventricles"[MeSH Terms] OR ("heart"[All Fields] AND "ventricles"[All Fields]) OR "heart ventricles"[All Fields] OR "ventricular"[All Fields]) AND ("arrhythmias, cardiac"[MeSH Terms] OR ("arrhythmias"[All Fields] AND "cardiac"[All Fields]) OR "cardiac arrhythmias"[All Fields] OR "arrhythmia"[All Fields]) AND ablation[All Fields]) AND (("2009/09/07"[PDAT] : "2019/09/07"[PDAT]) AND "humans"[MeSH Terms] AND English[lang])
      4. NOT editorial: 9164 remaining, 420 removed
         - ("tachycardia, ventricular"[MeSH Terms] OR ("tachycardia"[All Fields] AND "ventricular"[All Fields]) OR "ventricular tachycardia"[All Fields] OR ("ventricular"[All Fields] AND "tachycardia"[All Fields])) OR (("heart ventricles"[MeSH Terms] OR ("heart"[All Fields] AND "ventricles"[All Fields]) OR "heart ventricles"[All Fields] OR "ventricular"[All Fields]) AND ("arrhythmias, cardiac"[MeSH Terms] OR ("arrhythmias"[All Fields] AND "cardiac"[All Fields]) OR "cardiac arrhythmias"[All Fields] OR "arrhythmia"[All Fields]) AND ablation[All Fields]) NOT ("editorial"[Publication Type] OR "editorial"[All Fields]) AND (("2009/09/07"[PDAT] : "2019/09/07"[PDAT]) AND "humans"[MeSH Terms] AND English[lang])
      5. NOT letter: 8630 remaining, 534 removed
         - ("tachycardia, ventricular"[MeSH Terms] OR ("tachycardia"[All Fields] AND "ventricular"[All Fields]) OR "ventricular tachycardia"[All Fields] OR ("ventricular"[All Fields] AND "tachycardia"[All Fields])) OR (("heart ventricles"[MeSH Terms] OR ("heart"[All Fields] AND "ventricles"[All Fields]) OR "heart ventricles"[All Fields] OR "ventricular"[All Fields]) AND ("arrhythmias, cardiac"[MeSH Terms] OR ("arrhythmias"[All Fields] AND "cardiac"[All Fields]) OR "cardiac arrhythmias"[All Fields] OR "arrhythmia"[All Fields]) AND ablation[All Fields]) NOT (("editorial"[Publication Type] OR "editorial"[All Fields]) OR ("letter"[Publication Type] OR "correspondence as topic"[MeSH Terms] OR "letter"[All Fields])) AND (("2009/09/07"[PDAT] : "2019/09/07"[PDAT]) AND "humans"[MeSH Terms] AND English[lang])
      6. NOT review: 7050 remaining, 1580 removed
         - ("tachycardia, ventricular"[MeSH Terms] OR ("tachycardia"[All Fields] AND "ventricular"[All Fields]) OR "ventricular tachycardia"[All Fields] OR ("ventricular"[All Fields] AND "tachycardia"[All Fields])) OR (("heart ventricles"[MeSH Terms] OR ("heart"[All Fields] AND "ventricles"[All Fields]) OR "heart ventricles"[All Fields] OR "ventricular"[All Fields]) AND ("arrhythmias, cardiac"[MeSH Terms] OR ("arrhythmias"[All Fields] AND "cardiac"[All Fields]) OR "cardiac arrhythmias"[All Fields] OR "arrhythmia"[All Fields]) AND ablation[All Fields]) NOT (("editorial"[Publication Type] OR "editorial"[All Fields]) OR ("letter"[Publication Type] OR "correspondence as topic"[MeSH Terms] OR "letter"[All Fields]) OR ("review"[Publication Type] OR "review literature as topic"[MeSH Terms] OR "review"[All Fields])) AND (("2009/09/07"[PDAT] : "2019/09/07"[PDAT]) AND "humans"[MeSH Terms] AND English[lang])
      7. NOT case report: 4713 remaining, 2337 removed
         - ("tachycardia, ventricular"[MeSH Terms] OR ("tachycardia"[All Fields] AND "ventricular"[All Fields]) OR "ventricular tachycardia"[All Fields] OR ("ventricular"[All Fields] AND "tachycardia"[All Fields])) OR (("heart ventricles"[MeSH Terms] OR ("heart"[All Fields] AND "ventricles"[All Fields]) OR "heart ventricles"[All Fields] OR "ventricular"[All Fields]) AND ("arrhythmias, cardiac"[MeSH Terms] OR ("arrhythmias"[All Fields] AND "cardiac"[All Fields]) OR "cardiac arrhythmias"[All Fields] OR "arrhythmia"[All Fields]) AND ablation[All Fields]) NOT (("editorial"[Publication Type] OR "editorial"[All Fields]) OR ("letter"[Publication Type] OR "correspondence as topic"[MeSH Terms] OR "letter"[All Fields]) OR ("review"[Publication Type] OR "review literature as topic"[MeSH Terms] OR "review"[All Fields]) OR ("case reports"[Publication Type] OR "case report"[All Fields])) AND (("2009/09/07"[PDAT] : "2019/09/07"[PDAT]) AND "humans"[MeSH Terms] AND English[lang])
      8. NOT costs and costs analysis: 4696 remaining, 17 removed
         - ("tachycardia, ventricular"[MeSH Terms] OR ("tachycardia"[All Fields] AND "ventricular"[All Fields]) OR "ventricular tachycardia"[All Fields] OR ("ventricular"[All Fields] AND "tachycardia"[All Fields])) OR (("heart ventricles"[MeSH Terms] OR ("heart"[All Fields] AND "ventricles"[All Fields]) OR "heart ventricles"[All Fields] OR "ventricular"[All Fields]) AND ("arrhythmias, cardiac"[MeSH Terms] OR ("arrhythmias"[All Fields] AND "cardiac"[All Fields]) OR "cardiac arrhythmias"[All Fields] OR "arrhythmia"[All Fields]) AND ablation[All Fields]) NOT (("editorial"[Publication Type] OR "editorial"[All Fields]) OR ("letter"[Publication Type] OR "correspondence as topic"[MeSH Terms] OR "letter"[All Fields]) OR ("review"[Publication Type] OR "review literature as topic"[MeSH Terms] OR "review"[All Fields]) OR ("case reports"[Publication Type] OR "case report"[All Fields]) OR (("costs and cost analysis"[MeSH Terms] OR ("costs"[All Fields] AND "cost"[All Fields] AND "analysis"[All Fields]) OR "costs and cost analysis"[All Fields] OR "costs"[All Fields]) AND ("costs and cost analysis"[MeSH Terms] OR ("costs"[All Fields] AND "cost"[All Fields] AND "analysis"[All Fields]) OR "costs and cost analysis"[All Fields] OR "costs"[All Fields]) AND ("analysis"[Subheading] OR "analysis"[All Fields]))) AND (("2009/09/07"[PDAT] : "2019/09/07"[PDAT]) AND "humans"[MeSH Terms] AND English[lang])
2. VT[All Fields] AND ablation[All Fields]
   1. Results: 1934 references
      1. From 07/09/2009 to 07/09/2019: 1216 remaining, 718 removed
         - (vt[All Fields] AND ablation[All Fields]) AND ("2009/09/07"[PDAT] : "2019/09/07"[PDAT])
      2. English only: 1174 remaining, 42 removed
         - (vt[All Fields] AND ablation[All Fields]) AND (("2009/09/07"[PDAT] : "2019/09/07"[PDAT]) AND English[lang])
      3. Human studies only: 812 remaining, 362 removed
         - (vt[All Fields] AND ablation[All Fields]) AND (("2009/09/07"[PDAT] : "2019/09/07"[PDAT]) AND "humans"[MeSH Terms] AND English[lang])
      4. NOT editorial: 797 remaining, 15 removed
         - (vt[All Fields] AND ablation[All Fields]) NOT ("editorial"[Publication Type] OR "editorial"[All Fields]) AND (("2009/09/07"[PDAT] : "2019/09/07"[PDAT]) AND "humans"[MeSH Terms] AND English[lang])
      5. NOT letter: 794 remaining, 3 removed
         - (vt[All Fields] AND ablation[All Fields]) NOT (("editorial"[Publication Type] OR "editorial"[All Fields]) OR ("letter"[Publication Type] OR "correspondence as topic"[MeSH Terms] OR "letter"[All Fields])) AND (("2009/09/07"[PDAT] : "2019/09/07"[PDAT]) AND "humans"[MeSH Terms] AND English[lang])
      6. NOT review: 661 remaining, 133 removed
         - (vt[All Fields] AND ablation[All Fields]) NOT (("editorial"[Publication Type] OR "editorial"[All Fields]) OR ("letter"[Publication Type] OR "correspondence as topic"[MeSH Terms] OR "letter"[All Fields]) OR ("review"[Publication Type] OR "review literature as topic"[MeSH Terms] OR "review"[All Fields])) AND (("2009/09/07"[PDAT] : "2019/09/07"[PDAT]) AND "humans"[MeSH Terms] AND English[lang])
      7. NOT case report: 532 remaining, 129 removed
         - (vt[All Fields] AND ablation[All Fields]) NOT (("editorial"[Publication Type] OR "editorial"[All Fields]) OR ("letter"[Publication Type] OR "correspondence as topic"[MeSH Terms] OR "letter"[All Fields]) OR ("review"[Publication Type] OR "review literature as topic"[MeSH Terms] OR "review"[All Fields]) OR ("case reports"[Publication Type] OR "case report"[All Fields])) AND (("2009/09/07"[PDAT] : "2019/09/07"[PDAT]) AND "humans"[MeSH Terms] AND English[lang])
      8. NOT costs and costs analysis: 531 remaining, 1 removed
         - (vt[All Fields] AND ablation[All Fields]) NOT (("editorial"[Publication Type] OR "editorial"[All Fields]) OR ("letter"[Publication Type] OR "correspondence as topic"[MeSH Terms] OR "letter"[All Fields]) OR ("review"[Publication Type] OR "review literature as topic"[MeSH Terms] OR "review"[All Fields]) OR ("case reports"[Publication Type] OR "case report"[All Fields]) OR ("costs and cost analysis"[MeSH Terms] OR ("costs"[All Fields] AND "cost"[All Fields] AND "analysis"[All Fields]) OR "costs and cost analysis"[All Fields] OR "costs"[All Fields]) OR (("costs and cost analysis"[MeSH Terms] OR ("costs"[All Fields] AND "cost"[All Fields] AND "analysis"[All Fields]) OR "costs and cost analysis"[All Fields] OR "costs"[All Fields]) AND ("analysis"[Subheading] OR "analysis"[All Fields]))) AND (("2009/09/07"[PDAT] : "2019/09/07"[PDAT]) AND "humans"[MeSH Terms] AND English[lang])
3. ("catheter ablation"[MeSH Terms] OR ("catheter"[All Fields] AND "ablation"[All Fields]) OR "catheter ablation"[All Fields]) AND ("complications"[Subheading] OR "complications"[All Fields])
   1. Results: 12208 references
      1. From 07/09/2009 to 07/09/201: 6847 remaining, 5361 removed
         - (("catheter ablation"[MeSH Terms] OR ("catheter"[All Fields] AND "ablation"[All Fields]) OR "catheter ablation"[All Fields]) AND ("complications"[Subheading] OR "complications"[All Fields])) AND ("2009/09/07"[PDAT] : "2019/09/07"[PDAT])
      2. English only: 6328 remaining, 519 removed
         - (("catheter ablation"[MeSH Terms] OR ("catheter"[All Fields] AND "ablation"[All Fields]) OR "catheter ablation"[All Fields]) AND ("complications"[Subheading] OR "complications"[All Fields])) AND (("2009/09/07"[PDAT] : "2019/09/07"[PDAT]) AND English[lang])
      3. Human studies only: 5771 remaining, 557 removed
         - (("catheter ablation"[MeSH Terms] OR ("catheter"[All Fields] AND "ablation"[All Fields]) OR "catheter ablation"[All Fields]) AND ("complications"[Subheading] OR "complications"[All Fields])) AND (("2009/09/07"[PDAT] : "2019/09/07"[PDAT]) AND "humans"[MeSH Terms] AND English[lang])
      4. NOT editorial: 5651 remaining, 120 removed
         - (("catheter ablation"[MeSH Terms] OR ("catheter"[All Fields] AND "ablation"[All Fields]) OR "catheter ablation"[All Fields]) AND ("complications"[Subheading] OR "complications"[All Fields])) NOT ("editorial"[Publication Type] OR "editorial"[All Fields]) AND (("2009/09/07"[PDAT] : "2019/09/07"[PDAT]) AND "humans"[MeSH Terms] AND English[lang])
      5. NOT letter: 5478 remaining, 173 removed
         - (("catheter ablation"[MeSH Terms] OR ("catheter"[All Fields] AND "ablation"[All Fields]) OR "catheter ablation"[All Fields]) AND ("complications"[Subheading] OR "complications"[All Fields])) NOT (("editorial"[Publication Type] OR "editorial"[All Fields]) OR ("letter"[Publication Type] OR "correspondence as topic"[MeSH Terms] OR "letter"[All Fields])) AND (("2009/09/07"[PDAT] : "2019/09/07"[PDAT]) AND "humans"[MeSH Terms] AND English[lang])
      6. NOT review: 4215 remaining, 1263 removed
         - (("catheter ablation"[MeSH Terms] OR ("catheter"[All Fields] AND "ablation"[All Fields]) OR "catheter ablation"[All Fields]) AND ("complications"[Subheading] OR "complications"[All Fields])) NOT (("editorial"[Publication Type] OR "editorial"[All Fields]) OR ("letter"[Publication Type] OR "correspondence as topic"[MeSH Terms] OR "letter"[All Fields]) OR ("review"[Publication Type] OR "review literature as topic"[MeSH Terms] OR "review"[All Fields])) AND (("2009/09/07"[PDAT] : "2019/09/07"[PDAT]) AND "humans"[MeSH Terms] AND English[lang])
      7. NOT case report: 3301 remaining, 914 removed
         - (("catheter ablation"[MeSH Terms] OR ("catheter"[All Fields] AND "ablation"[All Fields]) OR "catheter ablation"[All Fields]) AND ("complications"[Subheading] OR "complications"[All Fields])) NOT (("editorial"[Publication Type] OR "editorial"[All Fields]) OR ("letter"[Publication Type] OR "correspondence as topic"[MeSH Terms] OR "letter"[All Fields]) OR ("review"[Publication Type] OR "review literature as topic"[MeSH Terms] OR "review"[All Fields]) OR ("case reports"[Publication Type] OR "case report"[All Fields])) AND (("2009/09/07"[PDAT] : "2019/09/07"[PDAT]) AND "humans"[MeSH Terms] AND English[lang])
      8. NOT costs and costs analysis: 3276 remaining, 25 removed
         - (("catheter ablation"[MeSH Terms] OR ("catheter"[All Fields] AND "ablation"[All Fields]) OR "catheter ablation"[All Fields]) AND ("complications"[Subheading] OR "complications"[All Fields])) NOT (("editorial"[Publication Type] OR "editorial"[All Fields]) OR ("letter"[Publication Type] OR "correspondence as topic"[MeSH Terms] OR "letter"[All Fields]) OR ("review"[Publication Type] OR "review literature as topic"[MeSH Terms] OR "review"[All Fields]) OR ("case reports"[Publication Type] OR "case report"[All Fields]) OR ("costs and cost analysis"[MeSH Terms] OR ("costs"[All Fields] AND "cost"[All Fields] AND "analysis"[All Fields]) OR "costs and cost analysis"[All Fields] OR "costs"[All Fields]) OR (("costs and cost analysis"[MeSH Terms] OR ("costs"[All Fields] AND "cost"[All Fields] AND "analysis"[All Fields]) OR "costs and cost analysis"[All Fields] OR "costs"[All Fields]) AND ("analysis"[Subheading] OR "analysis"[All Fields]))) AND (("2009/09/07"[PDAT] : "2019/09/07"[PDAT]) AND "humans"[MeSH Terms] AND English[lang])

# Study details

## Study characteristics

| Author | Year published | Multicentre/ single-centre | Number of patients | Study type | Quality score (1-8) |
| --- | --- | --- | --- | --- | --- |
| Wolf *et al*[1] | 2018 | Single-centre | 159 | Cohort | 7 |
| Siontis *et al*[2] | 2018 | Single-centre | 217 | Cohort | 6 |
| Pappone *et al*[3] | 2017 | Multicentre | 135 | Cohort | 4 |
| Kirubakaran *et al*[4] | 2017 | Single-centre | 29 | Cohort | 6 |
| Wei *et al*[5] | 2017 | Single-centre | 48 | Cohort | 5 |
| Nayyar *et al*[6] | 2017 | Single-centre | 27 | Case-controlled | 3 |
| Jin *et al*[7] | 2017 | Single-centre | 54 | Cohort | 8 |
| Kuroki *et al*[8] | 2017 | Single-centre | 109 | Cohort | 3 |
| Guo *et al*[9] | 2017 | Single-centre | 33 | Cohort | 3 |
| Kuck *et al*[10] | 2017 | Multicentre | 54 | Randomised controlled trial | 1 |
| Muser *et al*[11] | 2016 | Single-centre | 31 | Cohort | 6 |
| Lin *et al*[12] | 2016 | Single-centre | 70 | Cohort | 5 |
| Mussigbrodt *et al*[13] | 2016 | Single-centre | 45 | Cohort | 6 |
| Marchlinski *et al*[14] | 2016 | Multicentre | 233 | Cohort | 6 |
| Sapp *et al*[15] | 2016 | Multicentre | 132 | Randomised controlled trial | 8 |
| Skoda *et al*[16] | 2016 | Multicentre | 53 | Cohort | 5 |
| Di Biase *et al*[17] | 2016 | Multicentre | 118 | Randomised controlled trial | 4 |
| Souissi *et al*[18] | 2016 | Multicentre | 49 | Cohort | 3 |
| Fukunaga *et al*[19] | 2016 | Single-centre | 51 | Cohort | 5 |
| Muser *et al*[20] | 2016 | Single-centre | 282 | Cohort | 5 |
| Santangeli *et al*[21] | 2015 | Single-centre | 62 | Cohort | 5 |
| Acosta *et al*[22] | 2015 | Single-centre | 90 | Cohort | 4 |
| Izquierdo *et al*[23] | 2015 | Single-centre | 53 | Cohort | 1 |
| Luther *et al*[24] | 2015 | Single-centre | 24 | Cohort | 3 |
| Jin *et al*[25] | 2015 | Single-centre | 40 | Cohort | 5 |
| Suzuki *et al*[26] | 2015 | Single-centre | 42 | Cohort | 7 |
| Dinov *et al*[27] | 2014 | Single-centre | 55 | Cohort | 5 |
| Proietti *et al*[28] | 2014 | Single-centre | 142 | Cohort | 8 |
| Silberbauer *et al*[29] | 2014 | Single-centre | 160 | Cohort | 8 |
| Oloriz *et al*[30] | 2014 | Single-centre | 87 | Cohort | 6 |
| Chopra *et al*[31] | 2014 | Single-centre | 20 | Cohort | 5 |
| Fernandez-Armenta *et al*[32] | 2014 | Single-centre | 22 | Cohort | 6 |
| Mork *et al*[33] | 2014 | Single-centre | 98 | Cohort | 7 |
| Dinov *et al*[34] | 2013 | Single-centre | 227 | Cohort | 8 |
| Piers *et al*[35] | 2013 | Single-centre | 45 | Cohort | 7 |
| Pauriah *et al*[36] | 2013 | Single-centre | 45 | Cohort | 7 |
| Arenal *et al*[37] | 2012 | Single-centre | 59 | Cohort | 5 |
| Di Biase *et al*[38] | 2012 | Single-centre | 92 | Cohort | 5 |
| Philips *et al*[39] | 2012 | Single-centre | 87 | Cohort | 7 |
| Russo *et al*[40] | 2012 | Single-centre | 20 | Cohort | 6 |
| Kozluk *et al*[41] | 2011 | Single-centre | 24 | Cohort | 7 |
| Bai *et al*[42] | 2011 | Single-centre | 49 | Cohort | 5 |
| Alzand *et al*[43] | 2011 | Single-centre | 27 | Cohort | 6 |
| Silva *et al*[44] | 2011 | Single-centre | 26 | Cohort | 4 |
| Santangeli *et al*[45] | 2010 | Single-centre | 22 | Cohort | 6 |
| Deneke *et al*[46] | 2010 | Single-centre | 115 | Cohort | 6 |
| KÜhne *et al*[47] | 2010 | Single-centre | 35 | Cohort | 7 |
| Pluta *et al*[48] | 2010 | Single-centre | 21 | Cohort | 7 |
| Kuck *et al*[49] | 2010 | Multicentre | 52 | Randomised controlled trial | 7 |
| Tanner *et al*[50] | 2009 | Multicentre | 63 | Cohort | 7 |

# Additional analyses

## Pooled demographics and procedural characteristics

|  | Number of studies | Mean | SD | Minimum | Maximum |
| --- | --- | --- | --- | --- | --- |
| Males (%) | 48 | 81.5 | 14.4 | 30.6 | 99.6 |
| Age (years) | 50 | 58.7 | 10.8 | 34.0 | 72.0 |
| LV ejection fraction (%) | 45 | 36.5 | 9.7 | 21.0 | 57.0 |
| Underlying aetiology |  |  |  |  |  |
| ICM (%) | 50 | 63.2 | 47.3 | 0 | 100 |
| Total NICM (%) | 50 | 41.9 | 48.5 | 0 | 100 |
| ARVC (%) | 47 | 20.2 | 39.5 | 0 | 100 |
| DCM (%) | 47 | 7.2 | 20.7 | 0 | 100 |
| BrS (%) | 45 | 2.2 | 14.9 | 0 | 100 |
| Sarcoidosis (%) | 47 | 3.0 | 15.0 | 0 | 100 |
| Unspecified (%) | 49 | 11.5 | 28.2 | 0 | 100 |
| Previous CABG (%) | 25 | 23.7 | 21.4 | 0 | 62.5 |
| Diabetes (%) | 17 | 28.0 | 13.1 | 4.3 | 50.0 |
| Renal failure (%) | 9 | 16.2 | 9.8 | 0 | 34.5 |
| VT storm (%) | 28 | 49.3 | 32.4 | 0 | 100 |
| NYHA class III or IV (%) | 21 | 30.3 | 19.0 | 0 | 65.0 |
| Prior catheter VT ablation (%) | 33 | 31.2 | 44.1 | 0 | 100 |
| Procedure time (minutes) | 37 | 249.0 | 96.5 | 104.0 | 480 |
| Ablation time (minutes) | 26 | 36.2 | 26.0 | 9.0 | 114 |
| Ablation site (%) |  |  |  |  |  |
| LV only | 19 | 54.4 | 48.6 | 0 | 100 |
| RV only | 19 | 43.8 | 49.1 | 0 | 100 |
| Both LV and RV | 19 | 1.7 | 4.7 | 0 | 18.4 |
| Access site (%) |  |  |  |  |  |
| Endocardial only | 41 | 71.9 | 32.2 | 0 | 100 |
| Epicardial only | 42 | 1.9 | 9.2 | 0 | 59.1 |
| Combined endocardial/epicardial | 42 | 25.9 | 31.8 | 0 | 100 |
| Approach type (%) |  |  |  |  |  |
| Antegrade only | 12 | 34.2 | 42.4 | 0 | 100 |
| Retrograde only | 11 | 38.9 | 42.6 | 0 | 97.7 |
| Both ante- and retro-grade | 11 | 16.0 | 31.1 | 0 | 100 |
| Pre-procedural anticoagulation (%) | 19 | 97.9 | 9.2 | 60.0 | 100 |

ARVC, arrhythmogenic right ventricular cardiomyopathy; BrS, Brugada syndrome; CABG, coronary artery bypass graft; DCM, dilated cardiomyopathy; ICM, ischaemic cardiomyopathy; LV, left ventricular; NICM, non-ischaemic cardiomyopathy; NYHA, New York Heart Association; RV, right ventricular; SD, standard deviation; VT, ventricular tachycardia.

## Pooled major acute complications (n=50†)

|  | % pooled complication rate (95% CI) | I^2^ statistic (%) |
| --- | --- | --- |
| Any major acute complications (n=44)‡ | 8.83 (7.94 - 9.73) | 90 |
| Death (n=44)‡ | 0.85 (0.56 - 1.13) | 0 |
| Any vascular access-related complications | 1.95 (1.55 - 2.35) | 46 |
| Vascular access-related complications requiring intervention | 0.21 (0.08 - 0.35) | 0 |
| Any pericardial effusion | 1.44 (1.09 - 1.79) | 23 |
| Pericardial effusion requiring drainage | 0.86 (0.59 - 1.13) | 0 |
| Need for cardiac surgery | 0.31 (0.14 - 0.47) | 0 |
| Complete AV block | 0.38 (0.19 - 0.56) | 0 |
| CVA or TIA | 0.28 (0.13 - 0.44) | 0 |
| Cardiac perforation | 0.21 (0.08 - 0.35) | 0 |
| Cardiogenic shock or severe pulmonary oedema | 0.26 (0.11 - 0.41) | 0 |
| Lead displacement | 0.21 (0.08 - 0.35) | 0 |
| Venous or arterial thromboembolism | 0.21 (0.08 - 0.35) | 0 |
| MI | 0.14 (0.03 - 0.26) | 0 |
| Other major bleeding | 0.12 (0.02 - 0.22) | 0 |
| Infection requiring antibiotics | 0.05 (0 - 0.12) | 0 |
| Phrenic nerve palsy | 0.07 (0 - 0.12) | 0 |
| Pneumothorax or haemothorax | 0.07 (0 - 0.16) | 0 |

†unless otherwise stated

‡uncertain mortality rate in 6 studies, therefore excluded from analyses

AV, atrioventricular; CI, confidence interval; CVA, cerebrovascular accident; MI, myocardial infarction; TIA, transient ischaemic attack.

## Temporal trends of demographics and procedural characteristics in ICM

|  | 2009 - 2014 (n=13) | | | | 2014 - 2019 (n=16) | | | |  |
| --- | --- | --- | --- | --- | --- | --- | --- | --- | --- |
|  | Mean | | Range | | Mean | | Range | |  |
| Males (%) | 88.4 | | 71.4 - 100 | | 91.3 | | 87.0 - 99.6 | |  |
| Age (years) | 66.1 | | 62.0 - 70.0 | | 64.6 | | 37.0 - 72.0 | |  |
| LV ejection fraction (%) | 30.9 | | 26.0 - 40.0 | | 31.7 | | 21.0 - 49.0 | |  |
| Diabetes (%) | 21.7 | | 14.3 - 35.6 | | 33.7 | | 22.0 - 50.0 | |  |
| Renal failure (%) | 5.6 | | 0 - 11.1 | | 17.9 | | 12.1 - 22.6 | |  |
| VT storm (%) | 57.6 | | 0 - 100 | | 46.2 | | 12.1 - 100 | |  |
| NYHA class III or IV (%) | 22.0 | | 11.1 - 36.3 | | 36.4 | | 15.2 - 65.0 | |  |
| Prior catheter VT ablation (%) | 4.2 | | 0 - 25.0 | | 4.9 | | 0 - 29.6 | |  |
| Procedure time (minutes) | 203.3 | | 115.0 - 290.0 | | 245.3 | | 104.0 - 453.0 | |  |
| Ablation time (minutes) | 36.6 | | 11.0 - 76.0 | | 40.5 | | 9.0 - 114.0 | |  |
| Ablation site (%) |  | |  | |  | |  | |  |
| LV only | 100 | | 100 - 100 | | 96.4 | | 80.2 - 100 | |  |
| RV only | 0 | | 0 - 0 | | 0.2 | | 0 - 1.4 | |  |
| Both LV and RV | 0 | | 0 - 0 | | 3.4 | | 0 - 18.4 | |  |
| Access site (%) | |  | |  | |  | |  | |
| Endocardial only | 91.8 | | 48.9 - 100 | | 92.1 | | 70.0 - 100 | |  |
| Epicardial only | 0 | | 0 - 0 | | 0.1 | | 0 - 1.3 | |  |
| Combined endocardial/epicardial | 8.2 | | 0 - 51.1 | | 7.8 | | 0 - 30.0 | |  |
| Approach type (%) | |  | |  | |  | |  | |
| Antegrade only | 9.1 | | 0 - 28.6 | | 71.9 | | 0.9 - 100 | |  |
| Retrograde only | 61.4 | | 0 - 96.8 | | 23.4 | | 0 - 97.7 | |  |
| Both ante- and retro-grade | 29.5 | | 0 - 100 | | 4.4 | | 0 - 22.0 | |  |

ICM, ischaemic cardiomyopathy; LV, left ventricular; NYHA, New York Heart Association; RV, right ventricular; VT, ventricular tachycardia.

## Temporal trends of major acute complications in ICM

|  | 2009 - 2014 (n=13) | | 2014 - 2019 (n=16†) | |
| --- | --- | --- | --- | --- |
|  | % pooled complication rate (95% CI) | I^2^ statistic (%) | % pooled complication rate (95% CI) | I^2^ statistic (%) |
| Any major acute complications | 9.65 (7.47 - 11.83) | 89 | 9.19 (7.39 - 10.99)‡ | 91 |
| Death | 1.07 (0.42 - 1.71) | 0 | 0.81 (0.28 - 1.33)‡ | 0 |
| Any vascular access-related complication | 2.34 (1.35 - 3.32) | 51 | 2.66 (1.87 - 3.44) | 66 |
| Vascular access-related complication requiring intervention | 0.01 (0 - 0.06) | 0 | 0.35 (0.05 - 0.64) | 0 |
| Any pericardial effusion | 1.17 (0.49 - 1.85) | 2 | 1.84 (1.16 - 2.52) | 46 |
| Pericardial effusion requiring drainage | 0.43 (0.01 - 0.84) | 0 | 0.82 (0.36 - 1.28) | 0 |
| Need for cardiac surgery | 0.11 (0 - 0.32) | 0 | 0.34 (0.05 - 0.64) | 0 |
| Complete AV block | 0.53 (0.07 - 1.00) | 0 | 0.55 (0.17 - 0.92) | 0 |
| CVA or TIA | 0.75 (0.20 - 1.29) | 0 | 0.28 (0.01 - 0.54) | 0 |
| Cardiac perforation | 0.11 (0 - 0.32) | 0 | 0.21 (0 - 0.44) | 0 |
| Cardiogenic shock or severe pulmonary oedema | 0.64 (0.14 - 1.14) | 0 | 0.28 (0.01 - 0.54) | 0 |
| Lead displacement | 0.54 (0.08 - 0.99) | 0 | 0.28 (0.01 - 0.54) | 0 |
| Venous or arterial thromboembolism | 0.11 (0 - 0.32) | 0 | 0.01 (0 - 0.04) | 0 |
| MI | 0.22 (0 - 0.51) | 0 | 0.01 (0 - 0.04) | 0 |
| Other major bleeding | 0.01 (0 - 0.06) | 0 | 0.21 (0 - 0.44) | 0 |
| Infection requiring antibiotics | 0.11 (0 - 0.32) | 0 | 0.07 (0 - 0.21) | 0 |
| Phrenic nerve palsy | 0.01 (0 - 0.06) | 0 | 0.07 (0 - 0.21) | 0 |
| Pneumothorax or haemothorax | 0.11 (0 - 0.32) | 0 | 0.14 (0 - 0.33) | 0 |

†unless otherwise stated, ‡n=11

AV, atrioventricular; CI, confidence interval; CVA, cerebrovascular accident; MI, myocardial infarction; TIA, transient ischaemic attack.

## Temporal trends of demographics and procedural characteristics in NICM

|  | 2009 - 2014 (n=11) | | | | 2014 - 2019 (n=12) | | | |  |
| --- | --- | --- | --- | --- | --- | --- | --- | --- | --- |
|  | Mean | | Range | | Mean | | Range | |  |
| Males (%) | 68.6 | | 30.6 - 90.9 | | 74.2 | | 51.4 - 89.8 | |  |
| Age (years) | 50.2 | | 34.0 - 64.0 | | 50.8 | | 39.0 - 65.0 | |  |
| LV ejection fraction (%) | 42.6 | | 31.0 - 57.0 | | 44.7 | | 29.0 - 57.0 | |  |
| Diabetes (%) | 11.3 | | 11.1 - 11.5 | | 28.4 | | 4.3 - 42.9 | |  |
| Renal failure (%) | 21.7 | | 8.9 - 34.5 | | 20.2 | | 20.2 - 20.2 | |  |
| VT storm (%) | 47.4 | | 17.8 - 100 | | 52.8 | | 20.7 - 100 | |  |
| NYHA class III or IV (%) | 28.9 | | 0 - 50.6 | | 27.2 | | 2.0 - 54.8 | |  |
| Prior catheter VT ablation (%) | 20.3 | | 0 - 100 | | 26.4 | | 0 - 51.7 | |  |
| Procedure time (minutes) | 248.0 | | 161.0 - 375.0 | | 298.5 | | 169.0 - 480.0 | |  |
| Ablation time (minutes) | 29.7 | | 23.0 - 25.0 | | 33.1 | | 11.0 - 70.0 | |  |
| Ablation site (%) |  | |  | |  | |  | |  |
| LV only | 40.0 | | 0 - 100 | | 0 | | 0 - 0 | |  |
| RV only | 60.0 | | 0 - 100 | | 98.6 | | 91.3 - 100 | |  |
| Both LV and RV | 0 | | 0 - 0 | | 1.4 | | 0 - 8.7 | |  |
| Access site (%) | |  | |  | |  | |  | |
| Endocardial only | 41.0 | | 0 - 83.3 | | 56.8 | | 0 - 90.2 | |  |
| Epicardial only | 6.2 | | 0 - 59.1 | | 1.5 | | 0 - 10.6 | |  |
| Combined endocardial/epicardial | 48.6 | | 0 - 100 | | 41.8 | | 0 - 100 | |  |
| Approach type (%) | |  | |  | |  | |  | |
| Antegrade only | 0 | | 0 - 0 | | 5.2 | | 0 - 8.7 | |  |
| Retrograde only | 100 | | 100 - 100 | | 28.2 | | 0 - 56.3 | |  |
| Both ante- and retro-grade | 0 | | 0 - 0 | | 21.8 | | 0 - 43.7 | |  |

LV, left ventricular; NICM, non-ischaemic cardiomyopathy; NYHA, New York Heart Association; RV, right ventricular; VT, ventricular tachycardia.

## Temporal trends of major acute complications in NICM

|  | 2009 - 2014 (n=11†) | | 2014 - 2019 (n=12†) | |
| --- | --- | --- | --- | --- |
|  | % pooled complication rate (95% CI) | I^2^ statistic (%) | % pooled complication rate (95% CI) | I^2^ statistic (%) |
| Any major acute complications | 6.32 (3.75 - 8.89)‡ | 78 | 7.43 (6.05 - 8.82)^§^ | 92 |
| Death | 1.09 (0.15 - 2.04)‡ | 0 | 0.47 (0 - 0.84)^§^ | 0 |
| Any vascular access-related complication | 1.25 (0.27 - 2.23) | 0 | 1.13 (0.57 - 1.69) | 29 |
| Vascular access-related complication requiring intervention | 0.42 (0 - 1.00) | 0 | 0.15 (0 - 0.36) | 0 |
| Any pericardial effusion | 1.46 (0.41 - 2.51) | 0 | 1.20 (0.62 - 1.79) | 32 |
| Pericardial effusion requiring drainage | 1.04 (0.15 - 1.94) | 0 | 1.20 (0.62 - 1.79) | 32 |
| Need for cardiac surgery | 0.42 (0 - 1.00) | 0 | 0.38 (0.05 - 0.71) | 0 |
| Complete AV block | 0.63 (0 - 1.33) | 0 | 0 (0 - 0.04) | 0 |
| CVA or TIA | 0.01 (0 - 0.01) | 0 | 0.08 (0 - 0.23) | 0 |
| Cardiac perforation | 0.01 (0 - 0.10) | 0 | 0.38 (0.05 - 0.71) | 0 |
| Cardiogenic shock or severe pulmonary oedema | 0.01 (0 - 0.10) | 0 | 0.15 (0 - 0.36) | 0 |
| Lead displacement | 0.01 (0 - 0.10) | 0 | 0 (0 - 0.04) | 0 |
| Venous or arterial thromboembolism | 0.42 (0 - 1.00) | 0 | 0.45 (0.10 - 0.81) | 0 |
| MI | 0.22 (0 - 0.63) | 0 | 0.23 (0 - 0.48) | 0 |
| Other major bleeding | 0.01 (0 - 0.10) | 0 | 0.08 (0 - 0.23) | 0 |
| Infection requiring antibiotics | 0.01 (0 - 0.10) | 0 | 0 (0 - 0.04) | 0 |
| Phrenic nerve palsy | 0.22 (0 - 0.63) | 0 | 0.08 (0 - 0.23) | 0 |
| Pneumothorax or haemothorax | 0.01 (0 - 0.10) | 0 | 0 (0 - 0.04) | 0 |

†unless otherwise stated, ‡n=8, ^§^n=11

AV, atrioventricular; CI, confidence interval; CVA, cerebrovascular accident; MI, myocardial infarction; TIA, transient ischaemic attack.

## Major acute complications, prospective studies only (n=24†)

|  | % pooled complication rate (95% CI) | I^2^ statistic (%) |
| --- | --- | --- |
| Any major acute complications (n=22) | 6.52 (4.12 - 10.18) | 87 |
| Death (n=22) | 0.79 (0.34 - 1.83) | 46 |
| Any vascular access-related complications | 1.05 (0.49 - 2.21) | 62 |
| Vascular access-related complications requiring intervention | NA | NA |
| Any pericardial effusion | 1.07 (0.51 - 2.23) | 61 |
| Pericardial effusion requiring drainage | 0.42 (0.15 - 1.13) | 35 |
| Need for cardiac surgery | 0.08 (0.01 - 0.79) | 60 |
| Complete AV block | 0.42 (0.22 - 0.81) | 0 |
| CVA or TIA | 0.07 (0 - 1.11) | 77 |
| Cardiac perforation | 0.23 (0.10 - 0.56) | 0 |
| Cardiogenic shock or severe pulmonary oedema | 0.09 (0.01 - 1.35) | 69 |
| Lead displacement | 0.02 (0 - 1.14) | 83 |
| Venous or arterial thromboembolism | 0.05 (0.01 - 0.33) | 0 |
| MI | 0.14 (0.05 - 0.43) | 0 |
| Other major bleeding | 0.04 (0 - 1.10) | 64 |
| Infection requiring antibiotics | 0.05 (0.01 - 0.33) | 0 |
| Phrenic nerve palsy | NA | NA |
| Pneumothorax or haemothorax | 0.05 (0.01 - 0.33) | 0 |

†unless otherwise stated

AV, atrioventricular; CI, confidence interval; CVA, cerebrovascular accident; MI, myocardial infarction; RCT, randomised controlled trial; TIA, transient ischaemic attack.

## Major acute complications, RCTs only (n=4†)

|  | % pooled complication rate (95% CI) | I^2^ statistic (%) |
| --- | --- | --- |
| Any major acute complications (n=3) | 8.48 (3.10 - 13.86) | 45 |
| Death (n=3) | 0.01 (0 - 0.11) | 0 |
| Any vascular access-related complications | 1.05 (0.02 - 2.08) | 24 |
| Vascular access-related complications requiring intervention | 0.01 (0 - 0.08) | 0 |
| Any pericardial effusion | 1.84 (0.49 - 3.18) | 59 |
| Pericardial effusion requiring drainage | 0.53 (0 - 1.24) | 0 |
| Need for cardiac surgery | 0.01 (0 - 0.08) | 0 |
| Complete AV block | 0.53 (0 - 1.24) | 0 |
| CVA or TIA | 0.27 (0 - 0.78) | 0 |
| Cardiac perforation | 0.53 (0 - 1.25) | 0 |
| Cardiogenic shock or severe pulmonary oedema | 0.01 (0 - 0.08) | 0 |
| Lead displacement | 1.84 (0.52 - 3.15) | 60 |
| Venous or arterial thromboembolism | 0.01 (0 - 0.08) | 0 |
| MI | 0.27 (0 - 0.78) | 0 |
| Other major bleeding | 0.79 (0 - 1.67) | 1 |
| Infection requiring antibiotics | 0.01 (0 - 0.08) | 0 |
| Phrenic nerve palsy | 0.01 (0 - 0.08) | 0 |
| Pneumothorax or haemothorax | 0.01 (0 - 0.08) | 0 |

†unless otherwise stated

AV, atrioventricular; CI, confidence interval; CVA, cerebrovascular accident; MI, myocardial infarction; RCT, randomised controlled trial; TIA, transient ischaemic attack.

# Quality Assessment

Assessment of validity:

1. Are the inclusion and exclusion criteria clearly defined?
2. Was the selection of patients unbiased?
3. Were the reported complications specified and defined?
4. Was data on the complications prospectively collected?
5. Were the complications stratified?
6. Was the population studied described adequately?
7. Was the population studied representative of real world practice?
8. Did the study include a declaration of conflicts of interest and funding?

# References of Included Studies

1. Wolf, M., Sacher, F., Cochet, H., Kitamura, T., Takigawa, M., Yamashita, S., … Jaïs, P. (2018). Long-Term Outcome of Substrate Modification in Ablation of Post-Myocardial Infarction Ventricular Tachycardia. *Circulation: Arrhythmia and Electrophysiology*, *11*(2), 1–14. doi:10.1161/CIRCEP.117.005635

2. Siontis, K. C., Jamé, S., Sharaf Dabbagh, G., Latchamsetty, R., Jongnarangsin, K., Morady, F., & Bogun, F. M. (2018). Thromboembolic prophylaxis protocol with warfarin after radiofrequency catheter ablation of infarct-related ventricular tachycardia. *Journal of Cardiovascular Electrophysiology*, *29*(4), 584–590. doi:10.1111/jce.13418

3. Pappone, C., Brugada, J., Vicedomini, G., Ciconte, G., Manguso, F., Saviano, M., … Santinelli, V. (2017). Electrical Substrate Elimination in 135 Consecutive Patients with Brugada Syndrome. *Circulation: Arrhythmia and Electrophysiology*, *10*(5), 1–13. doi:10.1161/CIRCEP.117.005053

4. Kirubakaran, S., Bisceglia, C., Silberbauer, J., Oloriz, T., Santagostino, G., Yamase, M., … Della Bella, P. (2017). Characterization of the arrhythmogenic substrate in patients with arrhythmogenic right ventricular cardiomyopathy undergoing ventricular tachycardia ablation. *Europace*, *19*(6), 1049–1062. doi:10.1093/europace/euw062

5. Wei, W., Liao, H., Xue, Y., Fang, X., Huang, J., Liu, Y., … Wu, S. (2017). Long-term outcomes of radio-frequency catheter ablation on ventricular tachycardias due to arrhythmogenic right ventricular cardiomyopathy: A single center experience. *PLoS ONE*, *12*(1), 1–14. doi:10.1371/journal.pone.0169863

6. Nayyar, S., Wilson, L., Ganesan, A., Sullivan, T., Kuklik, P., Young, G., … Roberts-Thomson, K. C. (2018). Electrophysiologic features of protected channels in late postinfarction patients with and without spontaneous ventricular tachycardia. *Journal of Interventional Cardiac Electrophysiology*, *51*(1), 13–24. doi:10.1007/s10840-017-0299-6

7. Jin, Q., Jacobsen, P. K., Pehrson, S., & Chen, X. (2017). Prediction and prognosis of ventricular tachycardia recurrence after catheter ablation with remote magnetic navigation for electrical storm in patients with ischemic cardiomyopathy. *Clinical Cardiology*, *40*(11), 1083–1089. doi:10.1002/clc.22773

8. Kuroki, K., Nogami, A., Yoshida, K., Goya, M., Fukunaga, M., Kaitani, K., … Aonuma, K. (2017). Efficacy of Intensive Radiofrequency Energy Delivery to the Localized Dense Scar Area in Post-Infarction Ventricular Tachycardia Ablation - A Comparative Study With Standard Strategy Targeting the Infarcted Border Zone. *Circulation Journal*, *81*(11), 1603–1610. doi:10.1253/circj.cj-17-0306

9. Guo, J. R., Zheng, L. H., Wu, L. M., Ding, L. G., & Yao, Y. (2017). Aneurysm-related ischemic ventricular tachycardia: Safety and efficacy of catheter ablation. *Medicine*, *96*(13), 1–7. doi:10.1097/MD.0000000000006442

10. Kuck, K.-H., Tilz, R. R., Deneke, T., Hoffmann, B. A., Ventura, R., Hansen, P. S., … Willems, S. (2017). Impact of Substrate Modification by Catheter Ablation on Implantable Cardioverter–Defibrillator Interventions in Patients With Unstable Ventricular Arrhythmias and Coronary Artery Disease. *Circulation: Arrhythmia and Electrophysiology*, *10*(3), 1–9. doi:10.1161/circep.116.004422

11. Muser, D., Santangeli, P., Pathak, R. K., Castro, S. A., Liang, J. J., Magnani, S., … Marchlinski, F. E. (2016). Long-Term Outcomes of Catheter Ablation of Ventricular Tachycardia in Patients with Cardiac Sarcoidosis. *Circulation: Arrhythmia and Electrophysiology*, *9*(8), 1–13. doi:10.1161/CIRCEP.116.004333

12. Lin, C. Y., Chung, F. P., Lin, Y. J., Chang, S. L., Lo, L. W., Hu, Y. F., … Chen, S. A. (2017). Gender differences in patients with arrhythmogenic right ventricular dysplasia/cardiomyopathy: Clinical manifestations, electrophysiological properties, substrate characteristics, and prognosis of radiofrequency catheter ablation. *International Journal of Cardiology*, *227*, 930–937. doi:10.1016/j.ijcard.2016.11.055

13. Müssigbrodt, A., Efimova, E., Knopp, H., Bertagnolli, L., Dagres, N., Richter, S., … Arya, A. (2017). Should all patients with arrhythmogenic right ventricular dysplasia/cardiomyopathy undergo epicardial catheter ablation? *Journal of Interventional Cardiac Electrophysiology*, *48*(2), 193–199. doi:10.1007/s10840-016-0209-3

14. Marchlinski, F. E., Haffajee, C. I., Beshai, J. F., Dickfeld, T.-M. L., Gonzalez, M. D., Hsia, H. H., … Bhandari, A. K. (2016). Long-Term Success of Irrigated Radiofrequency Catheter Ablation of Sustained Ventricular Tachycardia: Post-Approval THERMOCOOL VT Trial. *Journal of the American College of Cardiology*, *67*(6), 674–683. doi:10.1016/j.jacc.2015.11.041

15. Sapp, J. L., Wells, G. A., Parkash, R., Stevenson, W. G., Blier, L., Sarrazin, J.-F., … Tang, A. S. (2016). Ventricular Tachycardia Ablation versus Escalation of Antiarrhythmic Drugs. *New England Journal of Medicine*, *375*(2), 111–121. doi:10.1056/NEJMoa1513614

16. Skoda, J., Arya, A., Garcia, F., Gerstenfeld, E., Marchlinski, F., Hindricks, G., … Reddy, V. Y. (2016). Catheter Ablation of Ischemic Ventricular Tachycardia with Remote Magnetic Navigation: STOP-VT Multicenter Trial. *Journal of Cardiovascular Electrophysiology*, *27*(October), S29–S37. doi:10.1111/jce.12910

17. Di Biase, L., Burkhardt, J. D., Lakkireddy, D., Carbucicchio, C., Mohanty, S., Mohanty, P., … Natale, A. (2015). Ablation of Stable VTs Versus Substrate Ablation in Ischemic Cardiomyopathy. *Journal of the American College of Cardiology*, *66*(25), 2872–2882. doi:10.1016/j.jacc.2015.10.026

18. Souissi, Z., Boulé, S., Hermida, J. S., Doucy, A., Mabo, P., Pavin, D., … Lacroix, D. (2018). Catheter ablation reduces ventricular tachycardia burden in patients with arrhythmogenic right ventricular cardiomyopathy: Insights from a north-western French multicentre registry. *Europace*, *20*(2), 362–369. doi:10.1093/europace/euw332

19. Fukunaga, M., Goya, M., Hiroshima, K., Hayashi, K., Ohe, M., Makihara, Y., … Iwabuchi, M. (2016). Impact of catheter ablation of ventricular tachycardia in patients with prior myocardial infarctions. *Journal of Arrhythmia*, *32*(6), 462–467. doi:10.1016/j.joa.2016.03.001

20. Muser, D., Santangeli, P., Castro, S. A., Pathak, R. K., Liang, J. J., Hayashi, T., … Marchlinski, F. E. (2016). Long-Term Outcome after Catheter Ablation of Ventricular Tachycardia in Patients with Nonischemic Dilated Cardiomyopathy. *Circulation: Arrhythmia and Electrophysiology*, *9*(10), 1–11. doi:10.1161/CIRCEP.116.004328

21. Santangeli, P., Zado, E. S., Supple, G. E., Haqqani, H. M., Garcia, F. C., Tschabrunn, C. M., … Marchlinski, F. E. (2015). Long-Term Outcome with Catheter Ablation of Ventricular Tachycardia in Patients with Arrhythmogenic Right Ventricular Cardiomyopathy. *Circulation: Arrhythmia and Electrophysiology*, *8*(6), 1413–1421. doi:10.1161/CIRCEP.115.003562

22. Acosta, J., Fernández-Armenta, J., Penela, D., Andreu, D., Borras, R., Vassanelli, F., … Berruezo, A. (2016). Infarct transmurality as a criterion for first-line endo-epicardial substrate-guided ventricular tachycardia ablation in ischemic cardiomyopathy. *Heart Rhythm*, *13*(1), 85–95. doi:10.1016/j.hrthm.2015.07.010

23. Izquierdo, M., Sánchez-Gómez, J. M., Ferrero De Loma-Osorio, A., Martínez, A., Bellver, A., Peláez, A., … Ruiz-Granell, R. (2015). Endo-Epicardial Versus Only-Endocardial Ablation as a First Line Strategy for the Treatment of Ventricular Tachycardia in Patients with Ischemic Heart Disease. *Circulation: Arrhythmia and Electrophysiology*, *8*(4), 882–889. doi:10.1161/CIRCEP.115.002827

24. Luther, V., Jamil-Copley, S., Koa-Wing, M., Shun-Shin, M., Hayat, S., Linton, N. W., … Kanagaratnam, P. (2015). Non-randomised comparison of acute and long-term outcomes of robotic versus manual ventricular tachycardia ablation in a single centre ischemic cohort. *Journal of Interventional Cardiac Electrophysiology*, *43*(2), 175–185. doi:10.1007/s10840-015-9992-5

25. Jin, Q., Jacobsen, P. K., Pehrson, S., & Chen, X. (2015). Acute and long term outcomes of catheter ablation using remote magnetic navigation for the treatment of electrical storm in patients with severe ischemic heart failure. *International Journal of Cardiology*, *183*(February 2014), 11–16. doi:10.1016/j.ijcard.2015.01.066

26. Suzuki, A., Yoshida, A., Takei, A., Fukuzawa, K., Kiuchi, K., Takami, K., … Hirata, K. I. (2015). Prophylactic catheter ablation of ventricular tachycardia before cardioverter-defibrillator implantation in patients with non-ischemic cardiomyopathy: Clinical outcomes after a single endocardial ablation. *Journal of Arrhythmia*, *31*(3), 122–129. doi:10.1016/j.joa.2014.09.007

27. Dinov, B., Schratter, A., Schirripa, V., Fiedler, L., Bollmann, A., Rolf, S., … Arya, A. (2015). Procedural Outcomes and Survival After Catheter Ablation of Ventricular Tachycardia in Relation to Electroanatomical Substrate in Patients With Nonischemic-Dilated Cardiomyopathy: The Role of Unipolar Voltage Mapping. *Journal of cardiovascular electrophysiology*, *26*(9), 985–993. doi:10.1111/jce.12715

28. Proietti, R., Essebag, V., Beardsall, J., Hache, P., Pantano, A., Wulffhart, Z., … Verma, A. (2015). Substrate-guided ablation of haemodynamically tolerated and untolerated ventricular tachycardia in patients with structural heart disease: Effect of cardiomyopathy type and acute success on long-term outcome. *Europace*, *17*(3), 461–467. doi:10.1093/europace/euu326

29. Silberbauer, J., Oloriz, T., Maccabelli, G., Tsiachris, D., Baratto, F., Vergara, P., … Bella, P. Della. (2014). Noninducibility and Late Potential Abolition. *Circulation: Arrhythmia and Electrophysiology*, *7*(3), 424–435. doi:10.1161/circep.113.001239

30. Oloriz, T., Silberbauer, J., MacCabelli, G., Mizuno, H., Baratto, F., Kirubakaran, S., … Bella, P. Della. (2014). Catheter ablation of ventricular arrhythmia in nonischemic cardiomyopathy: Anteroseptal versus inferolateral scar sub-types. *Circulation: Arrhythmia and Electrophysiology*, *7*(3), 414–423. doi:10.1161/CIRCEP.114.001568

31. Chopra, N., Tokuda, M., Ng, J., Reichlin, T., Nof, E., John, R. M., … Stevenson, W. G. (2014). Relation of the unipolar low-voltage penumbra surrounding the endocardial low-voltage scar to ventricular tachycardia circuit sites and ablation outcomes in ischemic cardiomyopathy. *Journal of Cardiovascular Electrophysiology*, *25*(6), 602–608. doi:10.1111/jce.12393

32. Fernandez-Armenta, J., Andreu, D., Penela, D., Trucco, E., Cipolletta, L., Arbelo, E., … Berruezo, A. (2014). Sinus rhythm detection of conducting channels and ventricular tachycardia isthmus in arrhythmogenic right ventricular cardiomyopathy. *Heart rhythm*, *11*(5), 747–754. doi:10.1016/j.hrthm.2014.02.016

33. Mørk, T. J., Kristensen, J., Gerdes, J. C., Jensen, H. K., Lukac, P., & Nielsen, J. C. (2014). Catheter ablation for ventricular tachycardia in ischaemic heart disease; Acute success and long-term outcome. *Scandinavian Cardiovascular Journal*, *48*(1), 27–34. doi:10.3109/14017431.2013.877154

34. Dinov, B., Fiedler, L., Schönbauer, R., Bollmann, A., Rolf, S., Piorkowski, C., … Arya, A. (2013). Outcomes in Catheter Ablation of Ventricular Tachycardia in Dilated Nonischemic Cardiomyopathy Compared With Ischemic Cardiomyopathy. *Circulation*, *129*(7), 728–736. doi:10.1161/circulationaha.113.003063

35. Piers, S. R. D., Leong, D. P., Van Huls Van Taxis, C. F. B., Tayyebi, M., Trines, S. A., Pijnappels, D. A., … Zeppenfeld, K. (2013). Outcome of ventricular tachycardia ablation in patients with nonischemic cardiomyopathy: The impact of noninducibility. *Circulation: Arrhythmia and Electrophysiology*, *6*(3), 513–521. doi:10.1161/CIRCEP.113.000089

36. Pauriah, M., Cismaru, G., Magnin-Poull, I., Andronache, M., Sellal, J. M., Schwartz, J., … De Chillou, C. (2013). A stepwise approach to the management of postinfarct ventricular tachycardia using catheter ablation as the first-line treatment: A single-center experience. *Circulation: Arrhythmia and Electrophysiology*, *6*(2), 351–356. doi:10.1161/CIRCEP.113.000261

37. Arenal, Á., Hernández, J., Calvo, D., Ceballos, C., Atéa, L., Datino, T., … Fernández-Avilés, F. (2013). Safety, long-term results, and predictors of recurrence after complete endocardial ventricular tachycardia substrate ablation in patients with previous myocardial infarction. *American Journal of Cardiology*, *111*(4), 499–505. doi:10.1016/j.amjcard.2012.10.031

38. Di Biase, L., Santangeli, P., Burkhardt, D. J., Bai, R., Mohanty, P., Carbucicchio, C., … Natale, A. (2012). Endo-epicardial homogenization of the scar versus limited substrate ablation for the treatment of electrical storms in patients with ischemic cardiomyopathy. *Journal of the American College of Cardiology*, *60*(2), 132–141. doi:10.1016/j.jacc.2012.03.044

39. Philips, B., Madhavan, S., James, C., Tichnell, C., Murray, B., Dalal, D., … Tandri, H. (2012). Outcomes of catheter ablation of ventricular tachycardia in arrhythmogenic right ventricular dysplasia/cardiomyopathy. *Circulation: Arrhythmia and Electrophysiology*, *5*(3), 499–505. doi:10.1161/CIRCEP.111.968677

40. Russo, A. Dello, Casella, M., Pieroni, M., Pelargonio, G., Bartoletti, S., Santangeli, P., … Tondo, C. (2012). Drug-refractory ventricular tachycardias after myocarditis: Endocardial and epicardial radiofrequency catheter ablation. *Circulation: Arrhythmia and Electrophysiology*, *5*(3), 492–498. doi:10.1161/CIRCEP.111.965012

41. Koźluk, E., Gaj, S., Kiliszek, M., Lodziński, P., Pia̧tkowska, A., & Opolski, G. (2011). Efficacy of catheter ablation in patients with an electrical storm. *Kardiologia Polska*, *69*(7), 665–670.

42. Bai, R., Di Biase, L., Shivkumar, K., Mohanty, P., Tung, R., Santangeli, P., … Natale, A. (2011). Ablation of Ventricular Arrhythmias in Arrhythmogenic Right Ventricular Dysplasia/Cardiomyopathy. *Circulation: Arrhythmia and Electrophysiology*, *4*(4), 478–485. doi:10.1161/CIRCEP.111.963066

43. Alzand, B. S. N., Timmermans, C. C. M. M., Wellens, H. J. J., Dennert, R., Philippens, S. A. M., Portegijs, P. J. M., & Rodriguez, L. M. (2011). Unmappable ventricular tachycardia after an old myocardial infarction. Long-term results of substrate modification in patients with an implantable cardioverter defibrillator. *Journal of Interventional Cardiac Electrophysiology*, *31*(2), 149–156. doi:10.1007/s10840-011-9549-1

44. Silva, G. G. da, Veloso, H. H., Leite, L. R., Farias, R. L., & Paola, A. A. V. de. (2011). Epicardial mapping of sustained ventricular tachycardia in nonischemic heart disease. *Arquivos brasileiros de cardiologia*, *96*(2), 114–120. doi:10.1590/s0066-782x2011005000009

45. Santangeli, P., Di Biase, L., Lakkireddy, D., Burkhardt, J. D., Pillarisetti, J., Michowitz, Y., … Natale, A. (2010). Radiofrequency catheter ablation of ventricular arrhythmias in patients with hypertrophic cardiomyopathy: Safety and feasibility. *Heart Rhythm*, *7*(8), 1036–1042. doi:10.1016/j.hrthm.2010.05.022

46. Deneke, T., Lawo, T., Grewe, P. H., Calcum, B., Rausse, R., Bösche, L., … Lemke, B. (2010). Usefulness of a Limited Linear Ablation of Post-Myocardial Infarction Ventricular Tachycardia Using a Standardized Approach Based on Sinus Rhythm Mapping. *American Journal of Cardiology*, *105*(9), 1235–1239. doi:10.1016/j.amjcard.2009.12.038

47. KÜhne, M., Abrams, G., Sarrazin, J. F., Crawford, T., Good, E., Chugh, A., … Bogun, F. M. (2010). Isolated potentials and pace-mapping as guides for ablation of ventricular tachycardia in various types of nonischemic cardiomyopathy. *Journal of Cardiovascular Electrophysiology*, *21*(9), 1017–1023. doi:10.1111/j.1540-8167.2010.01756.x

48. Pluta, S., Lenarczyk, R., Pruszkowska-Skrzep, P., Kowalski, O., Sokal, A., Sredniawa, B., … Kalarus, Z. (2010). Transseptal versus transaortic approach for radiofrequency ablation in patients with cardioverter-defibrillator and electrical storm. *Journal of Interventional Cardiac Electrophysiology*, *28*(1), 45–50. doi:10.1007/s10840-009-9464-x

49. Kuck, K.-H., Schaumann, A., Eckardt, L., Willems, S., Ventura, R., Delacretaz, E., … Hansen, P. S. (2010). Catheter ablation of stable ventricular tachycardia before defibrillator implantation in patients with coronary heart disease (VTACH): a multicentre randomised controlled trial. *Lancet*, *375*(9708), 31–40. doi:10.1016/S0140-6736(09)61755-4

50. Tanner, H., Hindricks, G., Volkmer, M., Furniss, S., KÜhlkamp, V., Lacroix, D., … Kottkamp, H. (2010). Catheter ablation of recurrent scar-related ventricular tachycardia using electroanatomical mapping and irrigated ablation technology: Results of the prospective multicenter Euro-VT-study. *Journal of Cardiovascular Electrophysiology*, *21*(1), 47–53. doi:10.1111/j.1540-8167.2009.01563.x
